# A multiepitope vaccine encoding four *Eimeria* epitopes with PLGA nanospheres: a novel vaccine candidate against coccidiosis in laying chickens

**DOI:** 10.1186/s13567-022-01045-w

**Published:** 2022-04-01

**Authors:** ZhengQing Yu, SiYing Chen, JianMei Huang, WenXi Ding, YuFeng Chen, JunZhi Su, RuoFeng Yan, LiXin Xu, XiaoKai Song, XiangRui Li

**Affiliations:** Ministry of Education (MOE) Joint International Research Laboratory of Animal Health and Food Safety, College of Veterinary Medicine, Nanjing Agricultural University, Nanjing, Jiangsu China

**Keywords:** *Eimeria* species, bioinformatics analysis, multiepitope vaccine, nanotechnology, immunogenicity, cross-protection

## Abstract

With a worldwide distribution, *Eimeria* spp. could result in serious economic losses to the poultry industry. Due to drug resistance and residues, there are no ideal drugs and vaccines against *Eimeria* spp. in food animals. In the current study, a bioinformatics approach was employed to design a multiepitope antigen, named NSLC protein, encoding antigenic epitopes of *E. necatrix* NA4, *E. tenella* SAG1, *E. acervulina* LDH, and *E. maxima* CDPK. Thereafter, the protective immunity of NSLC protein along with five adjuvants and two nanospheres in laying chickens was evaluated. Based on the humoral immunity, cellular immunity, oocyst burden, and the coefficient of growth, the optimum adjuvant was evaluated. Furthermore, the optimum immune route and dosage were also investigated according to the oocyst burden and coefficient of growth. Accompanied by promoted secretion of antibodies and enhanced CD4^+^ and CD8^+^ T lymphocyte proportions, NSLC proteins entrapped in PLGA nanospheres were more effective in stimulating protective immunity than other adjuvants or nanospheres, indicating that PLGA nanospheres were the optimum adjuvant for NSLC protein. In addition, a significantly inhibited oocyst burden and growth coefficient promotion were also observed in animals vaccinated with NSLC proteins entrapped in PLGA nanospheres, indicating that the optimum adjuvant for NSLC proteins was PLGA nanospheres. The results also suggested that the intramucosal route with PLGA nanospheres containing 300 μg of NSLC protein was the most efficient approach to induce protective immunity against the four *Eimeria* species. Collectively, PLGA nanospheres loaded with NSLC antigens are potential vaccine candidates against avian coccidiosis.

**Supplementary Information:**

The online version contains supplementary material available at 10.1186/s13567-022-01045-w.

## Introduction

Caused by single or multiple infections of *Eimeria* spp., avian coccidiosis is one of the most important intestinal diseases and can cost the poultry industry more than $3 billion annually [1, 2]. Due to the long-term existence of sporulated oocysts in the environment, *Eimeria* infection is very common in avian husbandry around the world [3]. Among the seven *Eimeria* spp., *Eimeria tenella* (*E. tenella*), *Eimeria necatrix* (*E. necatrix*), *Eimeria maxima* (*E. maxima*), and *Eimeria acervulina* (*E. acervulina*) are considered the main species that result in financial losses [1, 4]. Currently, *E. acervulina*, *E. maxima*, and *E. tenella* have entered a phase of high prevalence [5, 6], and *E. tenella* and *E. necatrix* are regarded as the most pathogenic. In addition, *E. acervulina* and *E. maxima* are usually less pathogenic but may cause intestinal malabsorption [7]. The transmission of *Eimeria* spp. can cause lower feed conversion ratios, poor growth, inferior laying performance, and even high mortality [8]. Anticoccidial drugs are considered the major effective way to control *Eimeria* infection. However, the increase in drug resistance and the chemical limits in food animals have forced the development of anti-coccidiosis vaccines [2].

Recently, novel strategies, including subunit and DNA vaccines, have been developed to control avian coccidiosis. Their applications in animals raise some difficulties, since subunit vaccines have poor reliability and may cause unexpected protective immunity [9], and DNA vaccines pose a theoretical risk of exogenous gene integration into the host genome. Multiepitope vaccines could conquer these limitations. Minimum antigenic epitopes are used to induce the expected immunoprotection and appear to be less likely to induce allergic reactions [10]. In addition, these strategies depend heavily on the protective antigens; thus, the identification of protective antigens is a key step in the development of *Eimeria* spp. vaccines.

Belonging to the Apicomplexa phylum, *Eimeria* spp. have secretory organelles, including micronemes (MICs), dense granules (GRAs), and rhoptries (ROPs). By secreting numerous secretory proteins, these secretory organelles play an essential role in regulating parasite invasion and survival [11]. As immunoproteomics methods have developed, a wide array of immunogenic antigens have been characterized in *Eimeria* sporozoites and merozoites [12]. Surface antigens (SAGs) are critical in parasite attachment and invasion and are used as vaccine candidates [13]. Derived from SAGs, *E. tenella* SAG1 (also known as TA4 antigen) can induce cell-mediated immunity against *E. tenella* infections [14]. Based on the nucleotide homology of *E. tenella* SAG1, NA4 antigens from *E. necatrix* have also been identified and can induce a strong protective effect against *E. necatrix* [15]. Exhibiting diverse kinetics and structures compared to their host, the lactate dehydrogenases (LDHs) of parasites are thus considered ideal targets for diagnosis and therapeutics [16]. DNA vaccines derived from *E. acervulina* LDH could inhibit the outputs of oocysts and enhance cell-mediated immunity, demonstrating inhibitory effects of *E. acervulina* [17]. Furthermore, previous studies have shown that calmodulin-domain protein kinases (CDPKs) are essential in *Eimeria* spp. infections [18]. Based on the vaccine antigens mentioned above, a multiepitope vaccine containing four different epitopes from different *Eimeria* spp. was constructed in the current study, and such a vaccine may prove to be a highly efficacious strategy in the development of vaccines against avian coccidiosis.

However, multiepitope vaccines are easily degraded by enzymes. Thus, an adjuvant is required to protect against undesirable degradation and to enhance immunogenicity [10]. Recently, nanospheres, as effective adjuvants to load antigens, have emerged as the most promising strategy to induce strong immunity [19]. Currently, many formulated nanospheres have shown great performance in anti-degradation and long-lasting humoral and cellular immunity induction [20, 21]. Widely applied in vaccines and drugs and approved by the FDA, polylactic-co-glycolic acid (PLGA) embodies many features, such as nontoxicity, good biocompatibility, and natural biodegradability [22]. Proven to be safe in the partial food industry, chitosan is also applied in biomedical materials mainly due to its biocompatible, biodegradable, and relatively safe properties [21].

In the current study, several bioinformatics tools were recruited to predict and design a multiepitope antigen, named NSLC protein, encoding the B- and T-cell epitopes of *E. necatrix* NA4 antigen (EnNA4), *E. tenella* surface antigen 1 (EtSAG1), *E. acervulina* lactate dehydrogenase (EaLDH), and *E. maxima* calmodulin-domain protein kinase (EmCDPK). With different adjuvants and nanomaterials, the NSLC proteins were encapsulated in five emulsions and two nanospheres. Then, the immune protection against coccidiosis induced by NSLC proteins and their encapsulations was investigated in laying chickens. In addition, we also optimized the procedures of NSLC proteins with optimum adjuvants, including the optimum immune route and optimum dosage.

## Materials and methods

### Poultry, cell lines, and parasites

Commercially available Hy-Line (new-born, breed W-36) were purchased from Shuangli Hatchery, Nantong, China, and were raised in coccidia-free conditions throughout the experiment. All animals had free access to coccidiostat-free food and water.

Purified oocysts of *E. tenella*, *E. acervulina*, *E. necatrix*, and *E. maxima* were preserved in the MOE Joint International Research Laboratory of Animal Health and Food Safety, College of Veterinary Medicine, Nanjing Agricultural University, Nanjing, China. Seven days prior to the challenge infection, the four types of *Eimeria* spp. oocysts used for challenge were propagated, collected, and sporulated as described previously [23].

### Bioinformatics prediction of candidate vaccine antigens

To obtain the potential epitopes, the amino acid sequences of EnNA4 (EU523548.1), EtSAG1 (AJ586531.2), EaLDH (FJ617009.1), and EmCDPK (Z71756.1) were retrieved from the NCBI database [24]. The antigenicity analysis was performed using DNAStar Protean (version 7.1.0, DNASTAR Inc., Madison, WI, USA). Based on the Berzofsky AMPHI [25] and the Rothbard-Taylor method [26] associated with the Jameson-Wolf algorithm [27], the T-cell epitopes and antigenic index were evaluated. The Hopp-Woods method [28] was also used to identify the hydrophilic regions. With a high antigenic index and hydrophilicity, amino acid sequences of potential T-cell epitopes were selected as the polypeptide antigen sequences.

### Assessment of secondary and tertiary structures

The secondary structure of the NSLC protein was predicted by the Garnier-Osguthorpe-Robson (GOR) IV online service [29]. Moreover, based on the multiple threading alignments and iterative template fragment assembly simulation methods, the I-TASSER server [30] was recruited to predict and assess the three-dimensional (3D) structure of the NSLC protein. All the parameters were set to default during the modelling of macromolecules. Then, PyMOL software (version 2.5, DeLano Scientific LLC, San Carlos, CA, USA) was employed to visualize the 3D structures.

### Prediction of transmembrane domains

The transmembrane domains of the NSLC protein were analysed by the online tool from the TMHMM server [31]. All the default parameters were used during the prediction.

### Prediction of antigenicity and allergenicity

According to a published paper [32], an online tool [33], known as Antigenic, was employed to evaluate the antigenicity of the NSLC protein. Based on recommended methods [34], the AlgPred 2.0 online tool [35] was subsequently used to estimate the allergenicity of the NSLC protein.

### Construction of the prokaryotic expression plasmid

According to the instructions, total RNA of 1 × 10^7^ sporulated oocysts of *E. tenella*, *E. acervulina*, *E. necatrix*, and *E. maxima* was isolated by using the Total RNA Extraction kit (OMEGA Bio-Tek, Norcross, GA, USA). To synthesize the cDNA, reverse transcription PCR (RT–PCR) was immediately carried out by using a reverse transcription kit (Takara Biotechnology, Dalian, China). Primers amplifying the polypeptide antigen sequences of EnNA4, EtSAG1, EaLDH, and EmCDPK were designed based on the conserved domain sequences (CDSs). Along with restriction endonuclease sites, the designed primers (Additional file 4) were synthesized by a company (Tsingke Biological Technology, Nanjing, China). PCR amplifications were conducted as follows: 1.25 U of *Ex Taq* DNA polymerase (Takara Biotechnology), 5 µL of 10× *Ex Taq* buffer, 4 µL of dNTP mixture (Mg^2+^ plus), 20 pmol of each primer, 2 ng of cDNA template, and ddH_2_O to make a final volume of 50 µL were used for each reaction. The amplification was determined by an amplifier (Thermo Scientific, Waltham, MA, USA) using the following program: preheating (5 min at 95 °C); amplification for 35 cycles of 30 s at 95 °C, 30 s at annealing temperatures and 70 s at 72 °C; and a final extension (5 min at 72 °C). Then, the PCR amplicons were confirmed (ABI PRISM™ 3730 XL DNA analyser, Applied Biosystems, Waltham, MA, USA), digested with restriction enzymes, and analysed in a 1.0% agarose gel. The fragments were cloned into the pET32a vector (Invitrogen Biotechnology, Shanghai, China) in accordance with the instructions of the T4 DNA ligase (Takara Biotechnology). The constructed plasmids were transferred to *Escherichia coli* (*E. coli*) BL21 (DE3) (Vazyme Biotech, Nanjing, China).

### Expression and purification of NSLC protein

Before protein expression, the recombinant plasmids were again verified by the ABI PRISM™ 3730 XL DNA analyser. According to the instructions, the NSLC protein was expressed under the induction of 1 mM isopropyl-β-d-thiogalactopyranoside (IPTG; Sigma–Aldrich, Shanghai, China) and purified with a chelating nickel column (Ni–NTA, GE Healthcare, Piscataway, NJ, USA). The purified protein was then analysed by 12% sodium salt polyacrylamide gel electrophoresis (SDS–PAGE) and stained with Coomassie blue. According to the directions of the ToxinEraser™ Endotoxin Removal Kit (GeneScript, Piscataway, NJ, USA), endotoxin was immediately removed after purification. The NSLC proteins were stored at −80 °C until use. Before further analysis, the concentrations and endotoxin levels of the NSLC protein were determined by a Pierce™ BCA Protein Assay Kit (Thermo Scientific) and ToxinSensor™ Chromogenic LAL Endotoxin Assay Kit (GeneScript, Piscataway, NJ, USA).

### Immunoblot analysis of NSLC protein

By oral administration, 14-day-old chickens without coccidia were challenged with sporulated oocysts of *E. tenella* (5 × 10^3^ oocysts per animal), *E. acervulina* (1 × 10^4^ oocysts per animal), *E. necatrix* (5 × 10^3^ oocysts per animal), or *E. maxima* (1 × 10^4^ oocysts per animal). With a seven-day interval, the booster challenges were conducted four times in total. Seven days after the last challenge, whole blood was collected from the wing vein of chickens, and the serum was then separated. Blank serum was also harvested from healthy animals without challenge. All sera were kept at −20 °C until use.

Following a previous study [36], Western blotting analysis was conducted with minor modifications. Briefly, the purified NSLC protein was first analysed in a 12% SDS–PAGE gel and then transferred to a polyvinylidene difluoride (PVDF) membrane (Immobilon-PSQ, Millipore, Billerica, MA, USA). Then, the blocking buffer, namely, TBST (TBS containing 0.5% Tween 20) containing 5% (*w*/*v*) skimmed milk powder, was used to block nonspecific binding sites at 37 °C for 2 h. Next, the membrane was rinsed in TBST for 5 min and incubated in chicken sera (1:100 dilution) at 37 °C for 2 h. After being washed three times in TBST, the membrane was then incubated with HRP-conjugated goat anti-rat IgG (1:5000 dilution, eBioscience, San Diego, USA) in TBST at 37 °C for 1 h. Finally, the membrane was visualized by the chemiluminescence ECL Western blotting analysis system (Tanon, Shanghai, China).

### Vaccine formulation

To synthesize the NSLC-FA emulsions (water-in-oil emulsion), complete Freund’s adjuvant (CFA, Sigma, Saint Louis, MO, USA) was added to an equal volume of NSLC protein solution. Then, the mixtures were placed on crushed ice, and tip sonication (Scientz Biotechnology, Ningbo, China) was conducted in continuous mode (durative time 3 s, interval time 3 s, 5 min in total) under an output power of 20 W (with 40% amplitude). The mixtures were inverted to mix thoroughly, and tip sonication was performed again. To avoid degradation, the synthesized NSLC-FA emulsions were stored at 4 °C for less than 2 h until use.

Based on a previous study [37], the double emulsion solvent evaporation technique (*w/o/w*) was conducted for the preparation of PLGA nanospheres with minor modifications. In brief, 50 mg of PLGA (MW: 40 000–75 000 Da, LA/GA: 65/35, Sigma) was first dissolved in 1 mL of dichloromethane (DCM, Sigma) to form the organic phase. At room temperature, 2 mL of 5% (*w/v*) polyvinyl alcohol (PVA, MW: 31 000–75 000 Da, Sigma) was set on a magnetic stirrer with constant stirring (200 rpm), and then 4 mL of NSLC protein (the concentration was 1 mg/mL) was added dropwise to synthesize the aqueous phase. The organic phase was added dropwise into the aqueous phase with a magnetic stirrer working at 400 rpm. To obtain the *w/o/w* emulsions, tip sonication was then conducted in continuous mode (durative time 5 s, interval time 5 s, 5 min in total) under an output power of 40 W in an ice bath. To remove DCM, the *w/o/w* emulsions were stirred at 400 rpm on a magnetic stirrer at room temperature for 2 h. The mixture was centrifuged at 35 000 rpm for 35 min at 4 °C, and the precipitates were collected and redissolved in double-distilled water. Before freezing at −80 °C for at least 2 h, the obtained PLGA nanospheres were passed through a 0.22-μm filter membrane (Millipore, Billerica, MA, USA), and then the frozen PLGA nanospheres were completely freeze-dried (Labconco, Kansas City, MO, USA). The NSLC-PLGA nanospheres were stored in powder form at 4 °C and diluted with PBS before use.

As described previously, the ionic gelation technique was conducted to prepare chitosan nanospheres [38]. Briefly, to prepare chitosan solution, 100 mg of chitosan (MW: 50–190 kDa, Sigma) was fully dissolved in 50 mL of 1% (*v/v*) aqueous solution of acetic acid, and the pH value was adjusted to 5.0 by NaOH solution. Four millilitres of 2 mg/mL sodium tripolyphosphate (TPP, Aladdin, Shanghai, China) solution was added dropwise into 20 mL of chitosan solution on a magnetic stirrer at a bath temperature of 30 °C. Subsequently, 4 mL of NSLC protein (the concentration was 1.0 mg/mL) was added dropwise. Next, tip sonication was performed in continuous mode (durative time 4 s, interval time 4 s, 3 min in total) under an output power of 50 W in an ice bath. After centrifugation at 35 000 rpm for 25 min at 4 °C, the precipitates were collected and redissolved in double-distilled water. Before freezing at −80 °C for at least 2 h, the obtained chitosan nanospheres were passed through a 0.22-μm filter membrane, and then the frozen chitosan nanospheres were completely freeze-dried. The NSLC-CS nanospheres were stored in powder form at 4 °C and diluted with PBS before use.

With minor modifications, the methods to synthesize the immune-stimulating complex (ISCOM) matrix have been described previously [39]. Briefly, 500 mg of N-decanoyl-N-methylglucamine (MEGA-10, Aladdin, Shanghai, China) was dissolved in 2.5 mL of double-distilled water to form a 20% (*w*/*v*) MEGA-10 solution. Then, 5 mg of cholesterol (Aladdin, Shanghai, China) and 5 mg of phosphatidylcholine (Aladdin, Shanghai, China) were mixed and dissolved in 500 μL of MEGA-10 solution to form the stock solution. Next, 1.3 mL of PBS and 10 mg of Quil A (a saponin-rich extract from *Quillaja saponaria*, CAS: 8047-15-2, Aladdin, Shanghai, China) were mixed with 200 μL of the stock solution. Tip sonication was performed in continuous mode (durative time 3 s, interval time 3 s, 10 min in total) under an output power of 40 W in an ice bath. The resulting mixture was dialyzed in PBS for 2 days by using a dialysis bag (molecular weight cut-off 7 kDa), and the PBS was changed every 8 h. The mixture after dialysis was diluted 1:10 with PBS and passed through a 0.22-μm filter membrane, and then the ISCOM matrix was obtained. To obtain the NSLC-ISCOM vaccines, the NSLC protein was first diluted in PBS (pH 7.4) to 1 mg/mL and then thoroughly mixed with an equal volume of ISCOM matrix by vortexing. To avoid degradation, the synthesized NSLC-ISCOM vaccines were stored at 4 °C for less than 2 h until use.

To synthesize the ginsenoside Rg1 solution, ginsenoside Rg1 (CAS: 22427-39-0, Aladdin, Shanghai, China) was first dissolved in a small amount of methanol (Aladdin, Shanghai, China) and then diluted to 30 mg/mL with PBS. To obtain the NSLC-RG1 vaccines, 1 mg/mL NSLC protein was mixed with an equal volume of ginsenoside Rg1 solution. To avoid degradation, the synthesized NSLC-RG1 vaccines were stored at 4 °C for less than 2 h until use.

For the NSLC-201VG emulsions (water-in-oil-in-water emulsion), Montanide™ ISA-201VG (Seppic, Paris, France) was prewarmed in a water bath (50 °C) for 10 min, and the NSLC protein was diluted in PBS (pH 7.4) to 1 mg/mL. In accordance with the instructions, the diluted protein was then gently added to Montanide™ ISA-201VG in a 1:1 (*w*/*w*) ratio at a bath temperature of 32 °C. The formulations were incubated for 10 min at a bath temperature of 32 °C with constant stirring (300 rpm). The synthesized NSLC-201VG emulsions were stored at 20 °C for less than 2 h until use.

To obtain the NSLC-71VR emulsions (water-in-oil emulsion), NSLC protein was added to Montanide™ ISA-71R VG (Seppic, Paris, France) at a ratio of 3:7 (*w*/*w*) with constant stirring for 15 min at room temperature, according to the manufacturer’s recommendations. The mixtures were then stirred by using an IKA T10 blender (IKA, Staufen, Germany) at 15 600 rpm for 10 min at room temperature until a homogenous emulsion was formed. To avoid degradation, the synthesized NSLC-71VR emulsions were stored at 4 °C for less than 2 h until use.

### Physical characterization of the synthesized nanospheres

To analyse the encapsulation efficiency (EE), the supernatant after ultracentrifugation was recycled, and the total volume was recorded. The concentrations of nonbound proteins in the supernatant were quantified by the Pierce™ BCA Protein Assay Kit. Then, the total amount of recombinant protein in the supernatant could be obtained, and the EE could be evaluated by Formula (1):

1 $${\text{Encapsulation efficiency }}\left( \% \right) = \frac{{{\text{Total protein}} - {\text{Free protein}}}}{{\text{Total protein}}} \times 100\%$$

To characterize the features of the NSLC-PLGA and NSLC-CS nanospheres, the nanospheres were sent to Nanjing Agriculture University for scanning electron microscope (SEM) observation with a Hitachi SU8010 (Tokyo, Japan). Based on the obtained images, the average diameter was assessed by measuring five arbitrary nanospheres using ImageJ software (version 1.8, NIH, Bethesda, MD, USA).

### Animal immunization and challenge schedules

New-born chickens were reared in coccidia-free environments and were randomly divided into eleven groups (30 animals/group). All animals were immunized intramuscularly in the leg muscles multiple times at seven-day intervals. For single vaccination, the vaccinated dosage of each animal did not exceed 500 μL. Animals were vaccinated with an equal volume of PBS that was used as a blank control (Table 1). At 28 days of age, animals in the experimental group were orally challenged with 5 × 10^4^ sporulated oocysts of *E. tenella*, while the animals in the blank (PBS) group were orally challenged with an equal volume of PBS. To evaluate the toxicity of different vaccines on normal animals, the chickens were observed every 12 h for physical health and mental status. Physical health mainly includes clinical symptoms at the injection site, daily diet consumption, and activity level, while mental status mainly includes stimulation reactions.

**Table 1:** **Vaccination strategies and challenge strategies**.

Group	Vaccination strategies		Challenge strategies		
	Treatment (each animal)	Vaccination time	Treatment (each animal)	Challenge time	Euthanasia time
Blank (PBS)	Equal volume of PBS	At 14 and 21 days of age	Equal volume of PBS (0 oocysts) at 28 days old	At 28 days of age	At 35 days of age, and the intestinal contents were separated
Blank (Coccidia)	Equal volume of PBS		5 × 10^4^ oocysts of *E. tenella* for each animal at 28 days old		
Control	200 μg of pET32a vector protein				
NSLC	200 μg of NSLC protein				
NSLC-FA	NSLC-FA emulsions containing 200 μg of NSLC protein				
NSLC-PLGA	NSLC-PLGA nanospheres containing 200 μg of NSLC protein				
NSLC-CS	NSLC-CS nanospheres containing 200 μg of NSLC protein				
NSLC-ISCOM	NSLC-ISCOM vaccines containing 200 μg of NSLC protein				
NSLC-RG1	NSLC-RG1 vaccines containing 200 μg of NSLC protein				
NSLC-201VG	NSLC-201VG emulsions containing 200 μg of NSLC protein				
NSLC-71VR	NSLC-71VR emulsions containing 200 μg of NSLC protein				

For body weight analysis, animals from each group were weighed at 14 (before the first immunization), 28 (before challenge), and 35 (seven days after challenge) days of age, and the coefficient of growth was evaluated according to Formula (2). Seven days after *E. tenella* infection (at 35 days of age), all the animals were euthanized under the supervision of the Animal Ethics Committee, Nanjing Agriculture University, China, at the same time, and the caecal contents were separated. According to McMaster’s counting technique, oocysts per gram (OPG) in caecal contents were investigated and are shown as the oocyst burden, and the oocyst reduction rate was also determined based on Formula (3).

2 $$\begin{aligned} & {\text{Coefficient of growth }}\left( \% \right) \\ &\quad= \frac{{{\text{Final weight}} - {\text{Initial weight}}}}{{\text{Initial weight}}} \times 100\% \end{aligned}$$

3 $${\text{Oocyst reduction }}\left( \% \right) = \frac{{{\text{OPG of Blank }}\left( {{\text{Coccidia}}} \right){\text{ group}} - {\text{OPG of experimental groups}}}}{{{\text{OPG of Blank }}\left( {{\text{Coccidia}}} \right){\text{ group}}}} \times 100\%$$

### Antibody and cytokine assays

At 14 (before the first immunization), 21 (seven days after the first immunization), and 28 (seven days after the second immunization) days of age, the animals were first anaesthetized, and blood was collected from the heart. The sera were then separated and stored at -20 °C until use. According to a previous study [14], enzyme-linked immunosorbent assay (ELISA) was performed to determine the NSLC-specific serum antibody levels. In brief, 96-well plates (Costar, Cambridge, MA, USA) were coated with 2 μg of NSLC protein (diluted to 20 μg/mL with carbonate buffer, pH 9.6) in each well overnight at 4 °C. After rinsing with TBST for 5 min, each well was blocked with TBS containing 5% (*w*/*v*) bovine serum albumin (BSA, Yifeixue, Nanjing, China) at 37 °C for 1 h. Then, the cells were incubated with chicken sera diluted 1:50 in TBS for 1 h at 37 °C after three washes in TBST. Subsequently, the cells were washed three times with TBST, and each well was incubated with HRP-conjugated anti-chicken IgY (1:8000, Abcam, Cambridge, UK) at 37 °C for 1 h to detect bound antibodies. Finally, 3,3′,5,5′-tetramethylbenzidine (TMB, Tiangen, Beijing, China) was used to develop the colour, and the reaction was stopped by 100 μL of 2 M newly prepared H_2_SO_4_. With a microplate photometer (Thermo Scientific), the absorbance was measured at 450 nm. Each group had five replications, and each replication was measured once.

Following the manufacturer’s instructions, the levels of cytokines in sera were assessed by commercially available ELISA kits (Enzyme-linked Biotechnology, Shanghai, China). The concentrations of interferon-gamma (IFN-γ), interleukin (IL) 4 (IL-4), transforming growth factor (TGF) β (TGF-β), and IL-17 were investigated based on the standard curves constructed from known amounts of chicken recombinant cytokines.

### Flow cytometry analysis

Fifteen animals in each group were euthanized at 21 (seven days after the first immunization) and 28 (seven days after the second immunization) days of age, and the splenic lymphocytes were separated as previously described [40]. The obtained lymphocytes were divided into two parts. To analyse the percentages of CD4^+^ T lymphocyte subsets, the splenic lymphocytes were stained with anti-chicken CD3e-FITC and CD4-PE (Southern Biotech, Birmingham, AL, USA) for 30 min at 4 °C in the dark. For the percentages of CD8^+^ T lymphocyte subsets, the splenic lymphocytes were stained with anti-chicken CD3e-FITC and CD8-PE (Southern Biotech) in the same environment. Before cell sorting by flow cytometry (Beckman Coulter Inc, Brea, CA, USA), lymphocytes were washed three times in PBS, and fluorescence compensation was carried out according to the instructions of CytExpert software (version 2.3, Beckman Coulter Inc, Brea, CA, USA). Each group had five replications, and each replication was assessed once.

### Optimum immune route and dosage

Based on the results of the experiments above, the optimum adjuvant was obtained. The effect of the optimum immune route on immune protection was assessed in vivo. New-born chickens were randomly assigned to six groups (10 animals/group). The immunization routes were intramuscular, intraoral, and intramucosal, and each route included blank (equal volume of PBS) and optimum adjuvant (vaccines containing 200 μg of NSLC protein) groups. For the intramuscular groups, immunization was conducted by the method described in the section “Animal immunization and challenge schedules”. For the intraoral groups, each animal was vaccinated by the intragastric method. For the intramucosal groups, vaccines were injected through the nose and eye drops. All infected animals were vaccinated using the same vaccine strategies as described in the section “Animal immunization and challenge schedules”. To evaluate the effect of the administration route on immune protection, animals were challenged intraorally with 5 × 10^4^ sporulated oocysts of *E. tenella* seven days after the last immunization, and the coefficient of growth, oocyst burden (expressed as OPG) in the caecal contents, and oocyst reduction rate were investigated.

According to the results of previous experiments, the optimum immune route was obtained. To evaluate the optimum immune dosage, new-born chickens were randomly divided into five groups (10 animals/group). The immunization dosages were the optimum vaccines containing 100 μg, 200 μg, 300 μg, or 400 μg of NSLC protein, and all infected animals were vaccinated using the same strategies as described in the section “Animal immunization and challenge schedules” through the optimum immune route. The same standards mentioned above were used to evaluate the effects of the administration dosage on immune protection.

### Cross-protection against four chicken coccidian species

New-born chickens were randomly assigned to eight groups (10 animals/group): the immunized group and the blank group challenged with four chicken coccidian species (*E. tenella*, *E. acervulina*, *E. necatrix*, and *E. maxima*). The adjuvant, immune route, and dosage were optimum, and the vaccine strategies were the same as those described in the section “Animal immunization and challenge schedules”. Seven days after the last immunization, animals were orally challenged with *E. tenella* (5 × 10^4^ sporulated oocysts per animal), *E. acervulina* (10 × 10^4^ sporulated oocysts per animal), *E. necatrix* (5 × 10^4^ sporulated oocysts per animal), and *E. maxima* (10 × 10^4^ sporulated oocysts per animal). Challenged animals were raised in different rooms under the same environmental conditions to avoid cross-infection. To illustrate the cross-protection of the vaccine containing NSLC protein, the coefficient of growth, oocyst burden (expressed as OPG) in the intestinal contents, and oocyst reduction rate were investigated. In addition, each *Eimeria* sp. had a particular predilection site in the gut. The predilection sites of *E. tenella* and *E. necatrix* were the caecum, while for *E. acervulina*, it was the duodenum, and for *E. maxima*, it was the mid-small intestine. The intestinal contents were collected for the oocyst burden according to the predilection sites.

### Data analysis

Significance analysis was evaluated by GraphPad software (Version 8.0, San Diego, CA, USA). One-way analysis of variance (ANOVA) was carried out on the levels of antibodies and cytokines, flow cytometry analysis, and oocyst burden in the intestinal contents. The independent *t* test was employed to analyse two groups of measurement values. Differences between groups were considered significant at *p* < 0.05, and values are expressed as the mean ± standard deviation (SD). Values for oocyst reduction are shown as the mean. Furthermore, the flow cytometric analysis was conducted by CytExpert software (version 2.3, Beckman Coulter Inc, Brea, CA, USA).

## Results

### Multiepitope vaccine design

The four specific candidate proteins as antigenic targets were analysed by DNAStar Protean software. Generally, the signal peptides can interfere with the expression of destined proteins; thus, the signal peptide sequences were removed by the PCR method. The selected sequences should contain potential epitopes with a high antigenic index and good hydrophilicity but should be as short as possible. Combined with two different models, potential epitopes were predicted (Additional file 1), including residues 21-123 (EnNA4), 36-144 (EtSAG1), 129-239 (EaLDH), and 191-292 (EmCDPK).

### Estimation of secondary and tertiary structures

**Figure 1:**
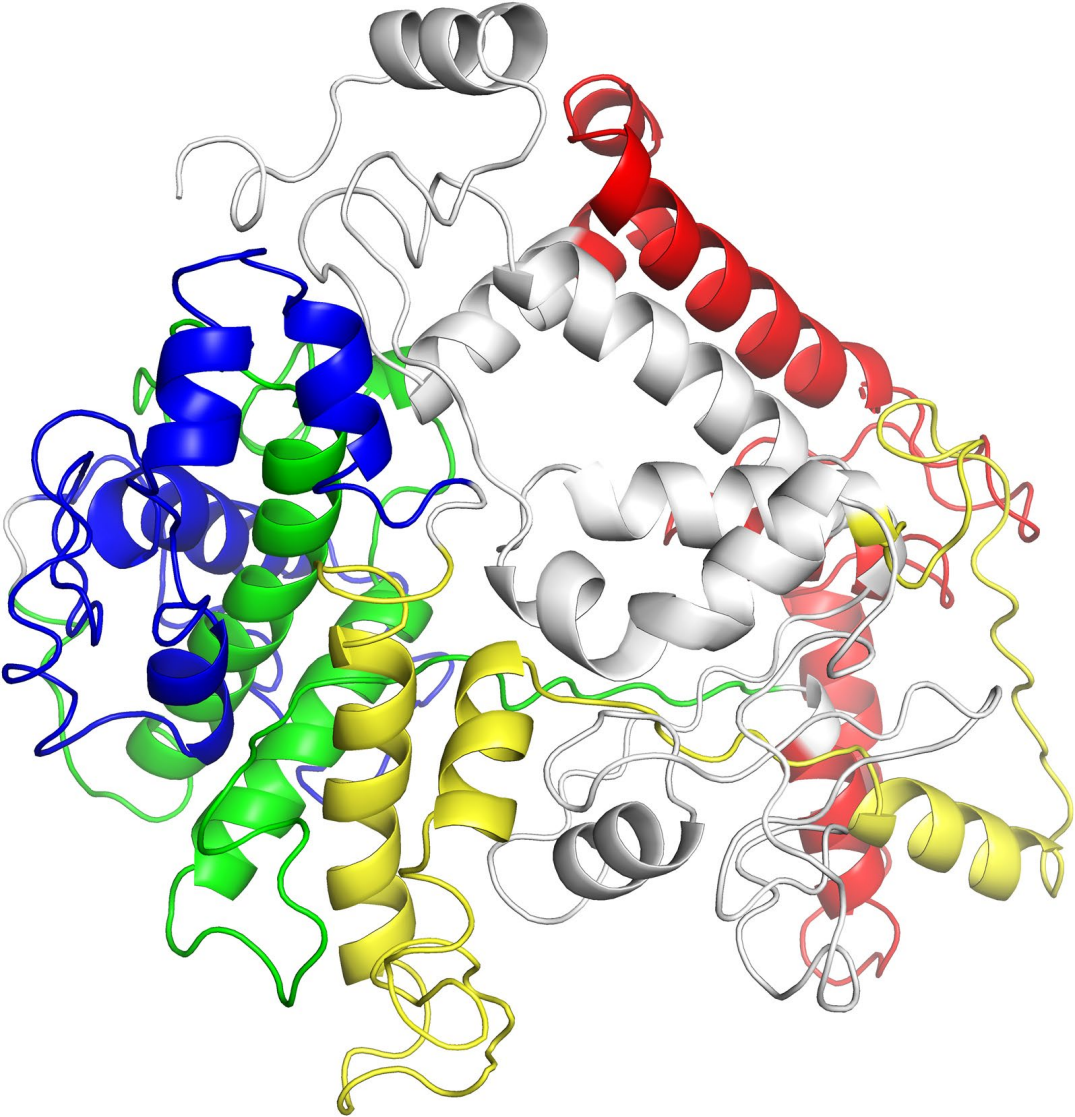
**Predicted 3D structure for the NSLC protein.** The 3D structure was predicted by the I-TASSER server and visualized by PyMOL software. White, red, green, blue, and yellow colours show the selected pET32a vector, EnNA4, EtSAG1, EaLDH, and EmCDPK proteins, respectively.

To estimate the secondary structure of the NSLC protein, the GOR IV online service was employed. The results indicated 12.11% extended strand, 45.50% random coil and 42.39% alpha-helix (Additional file 2). We used the I-TASSER server to estimate and assess the 3D model of the multiepitope antigen (Figure 1). Five 3D models of NSLC protein were predicted, and the model with highest C-score (−1.82) was selected (the range of the C-score is typically within −5 to 2, where the higher value indicates a higher confidence).

### Transmembrane domain, antigenicity, and allergenicity evaluation of NSLC protein

Based on the results of the TMHMM server, no transmembrane domain was observed in the NSLC protein (Additional file 3). The antigenic score of the NSLC protein was computed as 0.5415 using an online server, which demonstrated the antigenic nature of the NSLC protein. Furthermore, the AlgPred 2.0 online tool was employed to assess the allergenicity, and the nonallergic nature of NSLC protein was found.

### Cloning, expression, and purification of NSLC protein

**Figure 2:**
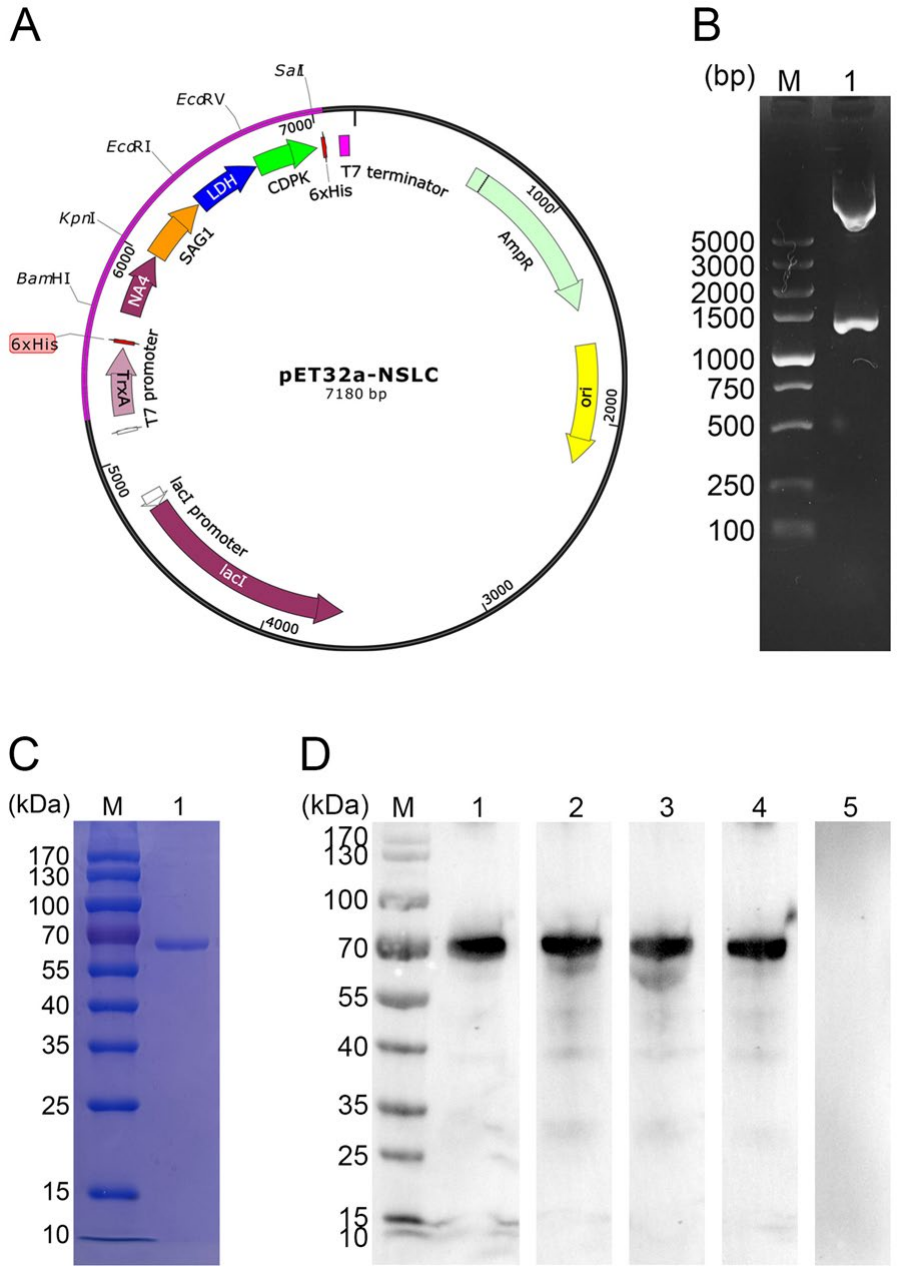
**Identification of the pET32a-NSLC vector and verification of the NSLC protein.** **A** Map of the constructed pET32a-NSLC vector containing the NSLC protein sequence. **B** Double digestion analysis of the constructed pET32a-NSLC vector. Lane M: DL5000 marker; Lane 1: Double digestion with *Bam*HI and *Sal*I. **C** SDS–PAGE analysis of purified NSLC protein (Lane 1). Lane M: MW marker proteins. **D** Western blot analysis of NSLC protein. Purified NSLC protein was detected in the sera from *E. tenella*-infected chickens (Lane 1), *E. acervulina*-infected chickens (Lane 2), *E. necatrix*-infected chickens (Lane 3), *E. maxima*-infected chickens (Lane 4), and healthy chickens (Lane 5). Lane M: MW marker proteins.

The recombinant plasmid pET32a-NSLC was successfully constructed as mentioned above (Figure 2A). To verify the recombinant plasmid, enzyme digestion was performed with *Bam*HI and *Sal*I, theoretically yielding two fragments of 1299 bp and 5881 bp (Figure 2B). The sequence analysis also confirmed that the insert in the recombinant plasmid was correct. All these results demonstrated that the recombinant plasmid was constructed correctly. The insert sequences encoded a fusion protein of 47.38 kDa. According to the guidelines, the recombinant protein expressed by the pET32a vector involved a His-tagged protein (19.10 kDa); therefore, the molecular weight of the fusion protein was 66.67 kDa in theory (Figure 2C). After removing the endotoxin from purified NSLC protein, the endotoxin level fell to 0.1 EU/mL.

### Immunoblot analysis of NSLC protein

Immunoblot analysis indicated that the NSLC proteins against *E. tenella*, *E. acervulina*, *E. necatrix*, and *E. maxima* could be detected in the sera from chickens (Figure 2D). All these results indicated that the antigenicity of the NSLC protein was satisfactory and could be detected by the host immune system.

### Physical characterization of the synthesized nanospheres

**Figure 3:**
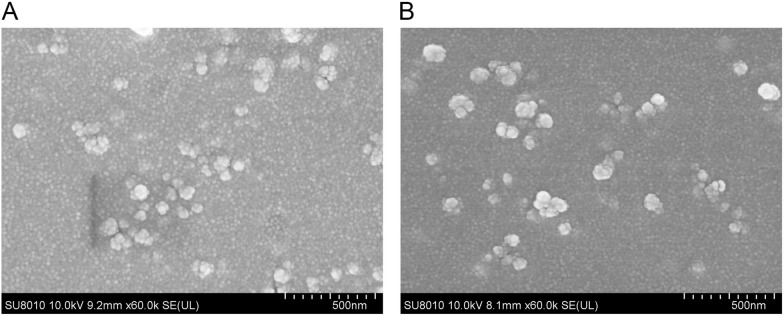
**SEM images of nanospheres loaded with NSLC protein.** **A** NSLC-PLGA nanospheres were formulated by the double emulsion solvent evaporation technique. **B** NSLC-CS nanospheres were synthesized by the ionic technique. SEM images showing the nanosphere morphology, with the bar representing 500 nm.

PLGA- and chitosan-encapsulated rTgPSA1 nanospheres were prepared by the double emulsion solvent evaporation technique and ionic gelation technique. The SEM images showed that both NSLC-PLGA (Figure 3A) and NSLC-CS nanospheres (Figure 3B) were spherical in shape with round convex particles on the surface. When the concentration of NSLC protein was 1 mg/mL, the EE was also analysed during the preparation, and the EE of NSLC-PLGA nanospheres reached 77.17% (*n* = 3), while the EE of NSLC-CS nanospheres reached 75.11% (*n* = 3). Based on the SEM images, the average diameter of NSLC-PLGA nanospheres was approximately 77.79 ± 7.86 nm (*n* = 5), while the mean diameter of NSLC-CS nanospheres was approximately 100.66 ± 15.49 nm (*n* = 5).

### Safety analysis of vaccination

During all in vivo experiments in our research, the health conditions of the animals were also evaluated through clinical observation. All animals were similar in physical health, and no abnormal changes occurred. All vaccinated animals were stable with regard to mental status during the trials compared with that of the animals from the blank group.

### Antibodies and cytokine production in animals

According to the standard ELISA procedure, the titres of total IgY in the sera collected from animals were investigated. As shown in Table 2, significantly increased levels of total IgY antibodies were found in the NSLC, NSLC-FA, NSLC-PLGA, NSLC-CS, NSLC-ISCOM, NSLC-201VG, and NSLC-71VR groups after the first immunization (21 days old). For the second immunization, all animals from the immunized groups revealed higher titres of IgY than the blank or control group. Furthermore, total IgY levels evaluated from the blank group were equal (*p* > 0.05) to those from the control group.

**Table 2:** **Antibody and cytokine determination in the sera from immunized chickens at 7, 14, and 21 days of age**.

Age	Group	Chicken IgY			Chicken IFN-γ			Chicken IL-4		
		Titre (OD450)	*p* value^a^	*p* value^b^	Value (pg/mL)	*p* value^a^	*p* value^b^	Value (pg/mL)	*p* value^a^	*p* value^b^
14 days	Blank (PBS)	0.180 ± 0.049	–	0.9721	29.541 ± 8.341	**–**	0.9968	83.088 ± 10.484	**–**	0.9977
	Control	0.202 ± 0.073	0.9721	–	27.372 ± 6.812	0.9968	**–**	79.879 ± 8.845	0.9977	**–**
	NSLC	0.158 ± 0.032	0.9721	0.5188	33.011 ± 8.623	0.9536	0.6451	79.365 ± 9.826	0.9969	>0.9999
	NSLC-FA	0.167 ± 0.030	0.9977	0.7529	29.564 ± 3.874	> 0.9999	0.9967	87.676 ± 11.376	0.9914	0.8428
	NSLC-PLGA	0.177 ± 0.051	0.9999	0.9427	31.261 ± 4.860	0.9994	0.9162	82.738 ± 12.697	>0.9999	0.9994
	NSLC-CS	0.187 ± 0.023	0.9997	0.9966	32.340 ± 3.372	0.9877	0.7639	80.648 ± 8.978	0.9995	0.9999
	NSLC-ISCOM	0.161 ± 0.033	0.9888	0.5913	29.492 ± 4.459	>0.9999	0.9969	85.182 ± 7.827	0.9996	0.9784
	NSLC-RG1	0.198 ± 0.040	0.9910	0.9998	30.208 ± 7.356	0.9998	0.9865	85.408 ± 9.470	0.9995	0.9721
	NSLC-201VG	0.204 ± 0.051	0.9542	>0.9999	32.229 ± 8.160	0.9903	0.7825	82.859 ± 9.487	>0.9999	0.9994
	NSLC-71VR	0.167 ± 0.029	0.9976	0.7478	31.224 ± 4.402	0.9994	0.9199	85.351 ± 17.905	0.9996	0.9738
21 days	Blank (PBS)	0.184 ± 0.041	–	0.9967	41.568 ± 8.879	**–**	0.9995	81.744 ± 8.473	**–**	0.9997
	Control	0.207 ± 0.067	0.9967	–	43.170 ± 6.579	0.9995	**–**	82.971 ± 9.361	0.9997	**–**
	NSLC	0.313 ± 0.036	0.0237	0.0877	55.939 ± 7.119	0.0295	0.0662	90.166 ± 13.679	0.7933	0.8931
	NSLC-FA	0.415 ± 0.047	<0.0001	<0.0001	59.452 ± 9.585	0.0041	0.0104	107.073 ± 8.736	0.0065	0.0105
	NSLC-PLGA	0.504 ± 0.055	<0.0001	<0.0001	64.417 ± 5.931	0.0002	0.0005	119.392 ± 12.301	<0.0001	<0.0001
	NSLC-CS	0.401 ± 0.068	<0.0001	0.0003	53.756 ± 4.372	0.0872	0.1763	125.150 ± 13.758	<0.0001	< 0.0001
	NSLC-ISCOM	0.306 ± 0.053	0.0374	0.1298	48.591 ± 3.741	0.5935	0.8279	139.137 ± 5.669	<0.0001	<0.0001
	NSLC-RG1	0.277 ± 0.041	0.1706	0.4424	61.762 ± 11.139	0.0010	0.0026	107.105 ± 11.632	0.0064	0.0104
	NSLC-201VG	0.366 ± 0.104	0.0007	0.0033	58.666 ± 6.583	0.0065	0.0161	131.842 ± 13.803	<0.0001	<0.0001
	NSLC-71VR	0.562 ± 0.102	<0.0001	<0.0001	62.424 ± 7.896	0.0007	0.0018	111.104 ± 10.736	0.0012	0.0021
28 days	Blank (PBS)	0.332 ± 0.051	–	0.9994	45.890 ± 4.798	**–**	0.9996	85.873 ± 9.740	**–**	0.9996
	Control	0.310 ± 0.125	0.9994	–	47.480 ± 6.860	0.9996	**–**	84.185 ± 6.600	0.9996	**–**
	NSLC	0.465 ± 0.069	0.1196	0.0497	63.593 ± 11.494	0.0271	0.0529	102.648 ± 8.300	0.0929	0.0519
	NSLC-FA	0.830 ± 0.065	<0.0001	<0.0001	65.113 ± 6.952	0.0137	0.0279	119.938 ± 12.526	<0.0001	<0.0001
	NSLC-PLGA	0.865 ± 0.089	<0.0001	<0.0001	71.442 ± 9.612	0.0006	0.0014	138.027 ± 12.043	<0.0001	<0.0001
	NSLC-CS	0.984 ± 0.082	<0.0001	<0.0001	64.268 ± 11.098	0.0201	0.0400	132.733 ± 15.030	< 0.0001	<0.0001
	NSLC-ISCOM	0.706 ± 0.095	<0.0001	<0.0001	64.909 ± 8.996	0.0151	0.0305	140.731 ± 8.930	<0.0001	<0.0001
	NSLC-RG1	0.635 ± 0.097	<0.0001	<0.0001	70.112 ± 7.111	0.0012	0.0027	116.959 ± 11.750	0.0003	0.0001
	NSLC-201VG	0.846 ± 0.104	<0.0001	<0.0001	67.100 ± 11.976	0.0054	0.0114	138.381 ± 8.845	<0.0001	<0.0001
	NSLC-71VR	1.097 ± 0.067	<0.0001	<0.0001	71.827 ± 9.695	0.0005	0.0011	131.663 ± 7.681	<0.0001	<0.0001

Age	Group	Chicken TGF-β			Chicken IL-17		
		Value (pg/mL)	*p* value^a^	*p* value^b^	Value (pg/mL)	*p* value^a^	*p* value^b^
14 days	Blank (PBS)	123.173 ± 12.248	**–**	0.9994	19.394 ± 5.137	**–**	0.9998
	Control	119.752 ± 8.148	0.9994	**–**	20.002 ± 7.549	0.9998	**–**
	NSLC	120.828 ± 15.092	0.9996	0.9998	20.369 ± 5.989	0.9996	0.9999
	NSLC-FA	114.260 ± 9.223	0.8371	0.9883	20.572 ± 6.462	0.9996	0.9998
	NSLC-PLGA	117.337 ± 13.643	0.9822	0.9996	20.533 ± 7.125	0.9996	0.9998
	NSLC-CS	110.500 ± 11.116	0.5066	0.8109	19.591 ± 5.885	>0.9999	0.9999
	NSLC-ISCOM	118.102 ± 12.177	0.9922	0.9997	21.239 ± 4.849	0.9975	0.9996
	NSLC-RG1	121.418 ± 11.854	0.9997	0.9997	19.883 ± 5.432	0.9998	>0.9999
	NSLC-201VG	117.947 ± 14.370	0.9911	0.9997	19.466 ± 4.633	>0.9999	0.9998
	NSLC-71VR	120.903 ± 15.142	0.9996	0.9998	18.501 ± 7.115	0.9997	0.9994
21 days	Blank (PBS)	121.005 ± 10.895	**–**	0.9997	23.460 ± 6.226	**–**	>0.9999
	Control	118.695 ± 12.262	0.9997	**–**	23.171 ± 4.923	>0.9999	**–**
	NSLC	127.328 ± 9.292	0.9902	0.9361	26.059 ± 4.507	0.9899	0.9794
	NSLC-FA	145.266 ± 12.469	0.0836	0.0475	32.456 ± 6.320	0.1396	0.1193
	NSLC-PLGA	135.244 ± 16.664	0.5657	0.3999	34.643 ± 6.006	0.0387	0.0322
	NSLC-CS	142.659 ± 22.132	0.1508	0.0897	32.020 ± 8.837	0.1752	0.1508
	NSLC-ISCOM	161.718 ± 16.989	0.0008	0.0004	29.559 ± 6.695	0.5142	0.4628
	NSLC-RG1	150.956 ± 8.537	0.0194	0.0101	36.107 ± 5.085	0.0147	0.0120
	NSLC-201VG	153.113 ± 17.809	0.0106	0.0054	31.193 ± 5.205	0.2625	0.2289
	NSLC-71VR	151.481 ± 15.511	0.0168	0.0087	26.425 ± 5.641	0.9758	0.9586
28 days	Blank (PBS)	124.590 ± 9.492	**–**	0.9999	26.130 ± 6.397	**–**	0.9993
	Control	123.421 ± 9.944	0.9999	**–**	28.280 ± 7.843	0.9993	**–**
	NSLC	139.386 ± 17.307	0.5903	0.5071	37.045 ± 6.409	0.1780	0.3860
	NSLC-FA	155.312 ± 15.596	0.0258	0.0191	42.596 ± 8.476	0.0126	0.0385
	NSLC-PLGA	158.200 ± 14.714	0.0122	0.0089	47.206 ± 8.857	0.0009	0.0031
	NSLC-CS	144.567 ± 10.419	0.2691	0.2173	49.501 ± 10.837	0.0002	0.0008
	NSLC-ISCOM	169.032 ± 15.898	0.0006	0.0004	44.582 ± 3.946	0.0041	0.0138
	NSLC-RG1	157.629 ± 16.096	0.0141	0.0103	49.309 ± 10.099	0.0003	0.0009
	NSLC-201VG	161.536 ± 22.182	0.0048	0.0035	40.471 ± 6.735	0.0381	0.1043
	NSLC-71VR	175.787 ± 20.563	<0.0001	<0.0001	42.661 ± 5.175	0.0122	0.0374

At 14 (before the first immunization), 21 (seven days after the first immunization), and 28 (seven days after the second immunization) days of age, sera were collected from five animals in each group. A standard ELISA procedure was carried out to investigate the titres of chicken IgY, and each sample was assessed once. Commercially available ELISA kits were used to determine the levels of IFN-γ, IL-4, TGF-β, and IL-17 in sera, and each sample was analysed once. The results were estimated using one-way ANOVA followed by Dunnett’s test, and the values are shown as the mean ± SD (*n* = 5).

^a, b^Were compared with the blank and control groups, respectively.

Strictly following the instructions, the sera collected from animals were used to evaluate the levels of IFN-γ, IL-4, IL-10, and IL-17. As illustrated in Table 2, obviously higher levels of IFN-γ could be detected in the NSLC, NSLC-FA, NSLC-PLGA, NSLC-RG1, NSLC-201VG, and NSLC-71VR groups at 21 days of age than in the blank and control groups. After the second immunization (28 days old), all immunized animals had higher levels of IFN-γ than the blank or control group. For IL-4, the secretions in immunized animals were significantly enhanced, except for the NSLC group after the first immunization (21 days old). Animals from all immunized groups generated higher levels of IL-4 after the second immunization (28 days old). For TGF-β, all animals except for those from the NSLC, NSLC-PLGA, and NSLC-CS groups generated significantly higher levels of TGF-β than the blank or control group at 21 days of age (after the first immunization). After the second vaccination (28 days old), enhanced secretion of TGF-β could be detected in all animals except for those from the NSLC and NSLC-CS groups. Compared to the blank or control group, obviously higher IL-17 secretion could be detected in the NSLC-PLGA and NSLC-RG1 groups at 21 days of age (after the first immunization). In addition, all animals except for those from the NSLC group were evaluated to have significantly higher levels of IL-17 after the second immunization (28 days old). Noticeably, comparisons between the blank and control groups were evaluated similarly (*p* > 0.05) for all investigated cytokines.

### Analysis of the cellular immune response in spleen lymphocytes

**Figure 4:**
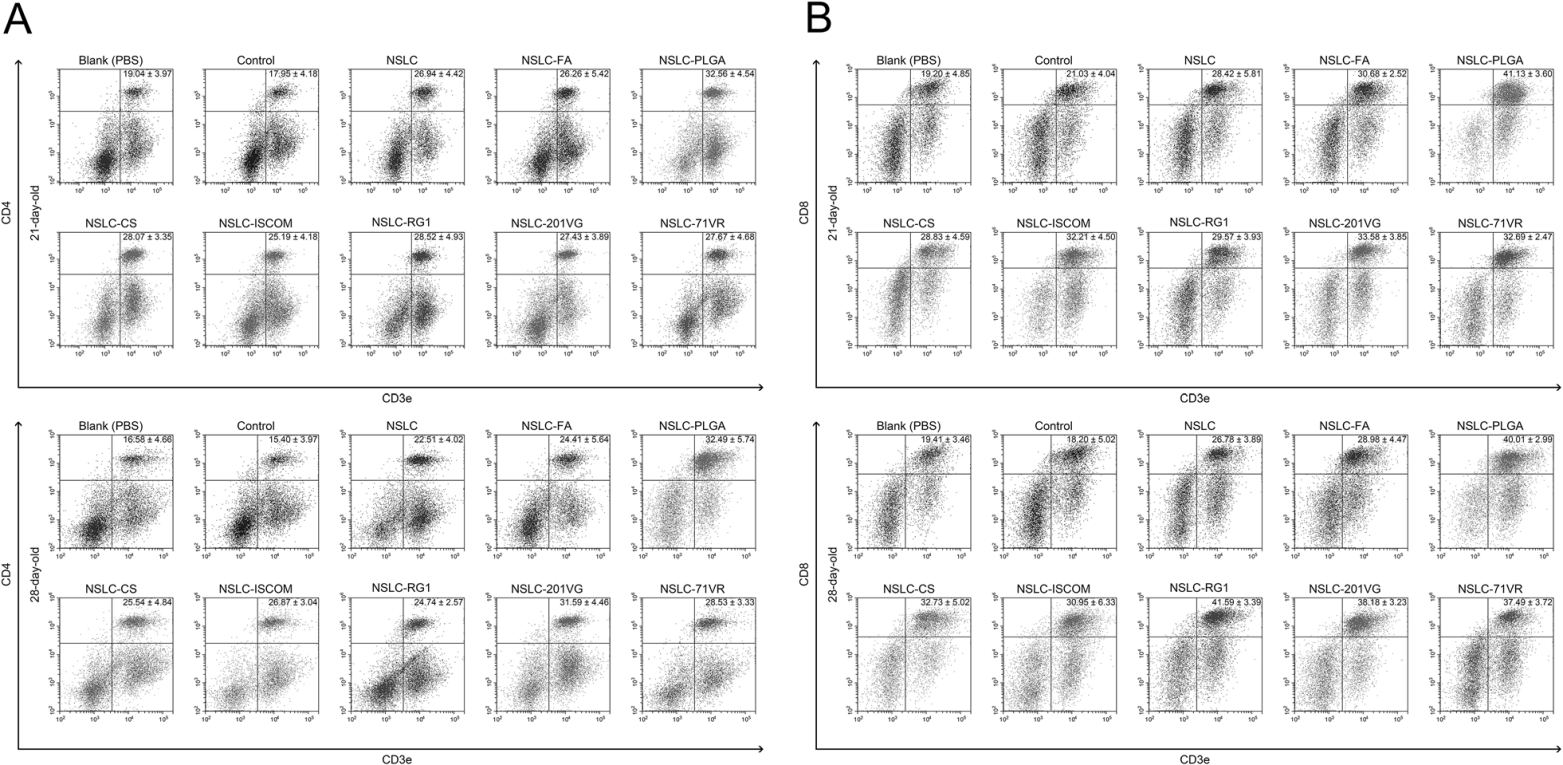
**Analysis of CD4**^**+**^** (A) and CD8**^**+**^** T lymphocytes (B) in spleen lymphocytes at 21 (seven days after the first immunization) and 28 (seven days after the second immunization) days of age by flow cytometry.** Five animals in each group were sacrificed, and splenic lymphocytes from each animal were collected. Each sample was assessed once, and values are shown as the mean ± SD (*n* = 5).

To determine the percentage of CD4^+^ and CD8^+^ T cells after immunization, five animals from each group were euthanized, and spleen lymphocytes were harvested. As illustrated in Figure 4A and Table 3, the percentage of CD4^+^ T cells in the NSLC, NSLC-FA, NSLC-PLGA, NSLC-CS, NSLC-RG1, NSLC-201VG, and NSLC-71VR groups was significantly increased after the first immunization (21 days old). One-way ANOVA also revealed that the NSLC-FA, NSLC-PLGA, NSLC-CS, NSLC-ISCOM, NSLC-RG1, NSLC-201VG, and NSLC-71VR groups were significantly enhanced after the second immunization (28 days old) when compared to the blank or control group. For the CD8^+^ T cells shown in Figure 4B and Table 3, increases could be detected in all immunized animals after the first (21-day-old) and second (28-day-old) immunizations. In addition, the blank group was statistically similar (*p* > 0.05) to the control group with respect to the investigated T lymphocytes.

**Table 3:** **Proportions of CD4**^**+**^** (a) and CD8**^**+**^** T lymphocytes in spleen lymphocytes at 14 and 21 days of age.**

Age	Group	CD4^+^ T lymphocytes			CD8^+^ T lymphocytes		
		Value (%)	*p* value ^a^	*p* value ^b^	Value (%)	*p* value ^a^	*p* value ^b^
21 days	Blank (PBS)	19.048 ± 3.970	–	0.9994	19.208 ± 4.855	–	0.9881
	Control	17.953 ± 4.186	0.9994	–	21.030 ± 4.049	0.9881	–
	NSLC	26.946 ± 4.420	0.0464	0.0173	28.425 ± 5.813	0.0080	0.0482
	NSLC-FA	26.260 ± 5.421	0.0821	0.0325	30.685 ± 2.528	0.0007	0.0051
	NSLC-PLGA	32.569 ± 4.541	0.0002	<0.0001	41.134 ± 3.606	<0.0001	<0.0001
	NSLC-CS	28.073 ± 3.350	0.0168	0.0058	28.830 ± 4.595	0.0052	0.0331
	NSLC-ISCOM	25.197 ± 4.180	0.1826	0.0800	32.210 ± 4.506	0.0001	0.0009
	NSLC-RG1	28.520 ± 4.933	0.0110	0.0037	29.571 ± 3.937	0.0023	0.0161
	NSLC-201VG	27.437 ± 3.891	0.0302	0.0109	33.581 ± 3.852	<0.0001	0.0002
	NSLC-71VR	27.675 ± 4.686	0.0243	0.0086	32.699 ± 2.478	<0.0001	0.0005
28 days	Blank (PBS)	16.583 ± 4.664	–	0.9994	19.410 ± 3.465	–	0.9993
	Control	15.400 ± 3.979	0.9994	–	18.207 ± 5.022	0.9993	–
	NSLC	22.514 ± 4.024	0.2037	0.0839	26.787 ± 3.892	0.0606	0.0204
	NSLC-FA	24.411 ± 5.647	0.0461	0.0157	28.981 ± 4.470	0.0077	0.0022
	NSLC-PLGA	32.497 ± 5.747	<0.0001	<0.0001	40.015 ± 2.996	<0.0001	<0.0001
	NSLC-CS	25.545 ± 4.842	0.0164	0.0051	32.733 ± 5.027	0.0001	<0.0001
	NSLC-ISCOM	26.876 ± 3.046	0.0044	0.0013	30.957 ± 6.338	0.0009	0.0003
	NSLC-RG1	24.743 ± 2.579	0.0344	0.0114	41.597 ± 3.392	<0.0001	<0.0001
	NSLC-201VG	31.594 ± 4.466	<0.0001	<0.0001	38.189 ± 3.237	<0.0001	<0.0001
	NSLC-71VR	28.536 ± 3.334	0.0008	0.0002	37.496 ± 3.729	<0.0001	<0.0001

At 21 (seven days after the first immunization) and 28 (seven days after the second immunization) days of age, five animals in each group were sacrificed, and splenic lymphocytes were collected. Each sample was assessed once, and the results were estimated by one-way ANOVA followed by Dunnett’s test and are shown as the mean ± SD (*n* = 5). ^a^ and ^b^ were compared with the blank and control groups, respectively.

### Determination of the optimum adjuvant

All animals were protected against *E. tenella* challenge and survived during the trials. The oocyst burden in the caecal contents was then evaluated, and all animals from the immunized group generated a significant (*p* < 0.001) inhibition compared to the blank and control groups (Table 4). Regarding oocyst reduction, immunized animals from the NSLC-ISCOM group showed the highest reduction rate (88.68%), followed by those from the NSLC-RG1 (82.85%) and the NSLC-PLGA (81.27%) groups. To evaluate the effects of different adjuvants on animal growth, the body weights were measured to evaluate the coefficient of growth. As evaluated in Table 4, the growth coefficients were significantly suppressed in animals from all immunized groups except for NSLC-201VG after *E. tenella* challenge (period of 28–35 days) when compared with the blank (PBS) group. Animals from the NSLC, NSLC-PLGA, NSLC-CS, NSLC-ISCOM, NSLC-RG1, and NSLC-201VG groups were observed to have markedly higher growth coefficients than those from the blank (coccidia) groups. In view of antibodies, cytokines, flow cytometry analysis, oocyst reduction, and the coefficient of growth, PLGA nanomaterial was considered to be the optimum adjuvant for NSLC proteins against *E. tenella*.

**Table 4:** **Immunoprotection of NSLC protein with different adjuvants**.

Group	Coefficient of growth (14–28 days of age)				Coefficient of growth (28–35 days of age)				Oocyst burden in caecal contents			Oocyst reduction (%)
	Value (%)	*p* value^a^	*p* value^b^	*p* value^c^	Value (%)	*p* value^a^	*p* value^b^	*p* value^c^	Value (× 10^6^, OPG)	*p* value^b^	*p* value^c^	
Blank (PBS)	113.470 ± 15.929	–	0.4639	0.2242	42.304 ± 10.695	–	<0.0001	<0.0001	–	–	–	–
Blank (Coccidia)	126.953 ± 24.954	0.4639	–	0.9994	18.506 ± 7.074	<0.0001	–	0.7142	10.960 ± 0.913	–	0.5322	–
Control	130.262 ± 19.041	0.2242	0.9994	–	23.762 ± 11.021	<0.0001	0.7142	–	10.498 ± 0.862	0.5322	–	4.214
NSLC	127.675 ± 23.559	0.4024	0.9999	0.9996	26.096 ± 11.340	<0.0001	0.2182	0.9987	6.438 ± 1.252	<0.0001	<0.0001	41.258
NSLC-FA	122.853 ± 31.616	0.8368	0.9992	0.9519	22.232 ± 8.078	<0.0001	0.9550	>0.9999	3.406 ± 1.769	<0.0001	<0.0001	68.925
NSLC-PLGA	115.997 ± 9.730	0.9996	0.7002	0.3977	31.690 ± 11.665	0.0173	0.0011	0.1721	2.053 ± 0.735	<0.0001	<0.0001	81.271
NSLC-CS	120.069 ± 41.894	0.9767	0.9693	0.7696	29.223 ± 10.961	0.0012	0.0156	0.6671	2.391 ± 0.554	<0.0001	<0.0001	78.182
NSLC-ISCOM	120.457 ± 38.920	0.9663	0.9793	0.8029	28.059 ± 11.272	0.0003	0.0463	0.8932	1.240 ± 0.597	<0.0001	<0.0001	88.688
NSLC-RG1	112.612 ± 11.816	0.9999	0.3916	0.1800	32.830 ± 10.204	0.0497	0.0003	0.0705	1.879 ± 0.593	<0.0001	<0.0001	82.854
NSLC-201VG	106.837 ± 10.315	0.9995	0.2479	0.1016	33.029 ± 10.650	0.0591	0.0002	0.0595	2.669 ± 0.806	<0.0001	<0.0001	75.651
NSLC-71VR	118.322 ± 25.292	0.9963	0.8898	0.6067	26.577 ± 12.282	<0.0001	0.1551	0.9941	2.671 ± 0.602	<0.0001	<0.0001	75.633

Twenty animals in each group were weighed at 14 (before the first immunization), 28 (before challenge), and 35 (seven days after challenge) days of age, and animals in each group were challenged with *E. tenella* at 28 days of age and euthanized seven days later. Caecal contents were collected to evaluate the effects of different adjuvants on the protective efficacy of NSLC proteins. Each sample was assessed once, and values were estimated by one-way ANOVA followed by Dunnett’s test and are shown as the mean ± SD (*n* = 20).

^a–c^Were compared with the blank (PBS), blank (coccidia), and control groups, respectively.

### Determination of the optimum immune route and dosage

New-born Hy-Line chickens were randomly divided into six groups, and all animals survived after the challenge infection. To illustrate the effect of the administration route on immune protection against *E. tenella*, the oocyst burden in the caecal contents and the coefficient of growth were investigated. Through three different immune routes (Table 5), all groups immunized with NSLC-PLGA nanospheres showed a remarkable oocyst decrease compared to that of the blank group. Animals immunized through the intramuscular route obtained a maximum reduction rate (75.61%), while the minimum rate (46.87%) was detected in animals vaccinated through the intraoral route. For the coefficient of growth, animals immunized by the intramucosal route could induce the highest level (37.38%) after *E. tenella* challenge (period of 28–35 days), while the lowest level was detected in animals immunized by the intraoral route (30.75%). Combined with the coefficient of growth and oocyst reduction, the obtained results suggested that the intramucosal route was the optimum immune route for NSLC-PLGA nanospheres.

**Table 5:** **The protective effects of NSLC-PLGA nanospheres through different immune routes**.

Immune route	Group	Coefficient of growth (14–28 days of age)		Coefficient of growth (28–35 days of age)		Oocyst burden in caecal contents		Oocyst reduction (%)
		Value (%)	*p* value	Value (%)	*p* value	Value (× 10^6^, OPG)	*p* value	
Intramuscular	NSLC-PLGA	140.515 ± 19.453	0.4876	36.483 ± 14.973	0.0036	0.905 ± 0.336	<0.0001	75.614
	Blank	130.595 ± 39.764	–	18.510 ± 8.074	–	3.710 ± 0.930	–	–
Intraoral	NSLC-PLGA	129.593 ± 14.330	0.2116	30.756 ± 21.551	0.1458	2.198 ± 0.417	<0.0001	46.871
	Blank	116.634 ± 28.205	–	19.535 ± 8.958	–	4.137 ± 0.899	–	–
Intramucosal	NSLC-PLGA	122.197 ± 43.538	0.6250	37.386 ± 8.511	0.0003	1.326 ± 0.360	<0.0001	65.520
	Blank	111.758 ± 50.104	–	21.388 ± 7.488	–	3.847 ± 0.760	–	–

Ten animals in each group were weighed at 14 (before the first immunization), 28 (before challenge), and 35 (seven days after challenge) days of age, and animals in each group were challenged with *E. tenella* at 28 days of age and euthanized seven days later. The caecal contents were collected to evaluate the effects of different immune routes on the protective efficacy of NSLC-PLGA nanospheres. Each sample was assessed once, and values were estimated by the independent *t* test. Values of oocyst burden are shown as the mean ± SD (*n* = 10). *p* values were obtained by comparison with the blank group.

To investigate the optimum immune dosage, all animals were immunized through the intramucosal route, and the survival rate was 100%. As illustrated in Table 6, a significant (*p* < 0.001) decrease in *E. tenella* oocysts was detected in all experimental animals after immunizations with different dosages of NSLC-PLGA nanospheres. Furthermore, the maximum and minimum oocyst reduction rates were detected in the Mucosa-400 (80.81%) and Mucosa-200 (68.48%), groups, respectively. Regarding the coefficient of growth after *E. tenella* challenge (period of 28–35 days, Table 6), statistically (*p* < 0.001) higher levels were observed in animals immunized with nanospheres containing 200 μg, 300 μg, or 400 μg of NSLC protein than in the blank group. Collectively, the results suggested that NSLC-PLGA nanospheres containing 200 μg, 300 μg or 400 μg of NSLC protein were efficient in resisting *E. tenella*. By generating the highest coefficient of growth, PLGA nanospheres containing 300 μg of NSLC protein were regarded as the optimum.

**Table 6:** **Immunoprotection with different immune dosages**.

Group	Coefficient of growth (14–28 days of age)		Coefficient of growth (28–35 days of age)		Oocyst burden in caecal contents		Oocyst reduction (%)
	Value (%)	*p* value	Value (%)	*p* value	Value (× 10^6^, OPG)	*p* value	
Blank	131.771 ± 17.237	–	19.447 ± 5.140	–	3.585 ± 0.316	–	–
Mucosa-100	127.427 ± 42.997	0.9993	18.623 ± 4.598	0.9975	0.951 ± 0.184	<0.0001	73.486
Mucosa-200	135.419 ± 67.403	0.9998	39.854 ± 11.459	<0.0001	1.130 ± 0.213	<0.0001	68.483
Mucosa-300	154.931 ± 92.832	0.8056	40.750 ± 6.854	<0.0001	0.821 ± 0.157	<0.0001	77.112
Mucosa-400	112.170 ± 54.163	0.8793	38.501 ± 8.069	<0.0001	0.688 ± 0.379	<0.0001	80.811

Ten animals in each group were weighed at 14 (before the first immunization), 28 (before challenge), and 35 (seven days after challenge) days of age, and animals in each group were challenged with *E. tenella* at 28 days of age and euthanized seven days later. The caecal contents were collected to evaluate the effects of different dosages of NSLC-PLGA nanospheres on immunoprotection. Each sample was assessed once, and the oocyst burden in the caecal contents was estimated by one-way ANOVA followed by Dunnett’s test. Values of oocyst burden are shown as the mean ± SD (*n* = 10). *p* values were obtained by comparison with the blank group.

### Cross-protection against four *Eimeria* spp.

To demonstrate the cross-protection of NSLC-PLGA nanospheres, animals were grouped and then challenged with *E. tenella*, *E. acervulina*, *E. necatrix*, and *E. maxima*. All immunized animals survived after the challenge. When challenged with the four *Eimeria* spp., the vaccinated animals generated a significant decrease in oocyst burden (Table 7). Moreover, the oocyst reduction rate ranged from 74.65% to 88.18%, and the maximum rate occurred in animals challenged with *E. necatrix*, while the minimum rate was detected in animals challenged with *E. maxima*. For the coefficient of growth summarized in Table 7, all immunized animals were significantly protected after *Eimeria* challenge (period of 28–35 days) according to the independent *t* test. Overall, NSLC-PLGA nanospheres containing 300 μg of NSLC protein generated satisfactory immunoprotection against four *Eimeria* spp.

**Table 7:** **Cross-protection of NSLC-PLGA nanospheres against four** ***Eimeria*** **spp**.

Challenged *Eimeria* spp.	Group	Coefficient of growth (14–28 days of age)		Coefficient of growth (28–35 days of age)		Oocyst burden in intestinal contents		Oocyst reduction (%)
		Value (%)	*p* value	Value (%)	*p* value	Value (× 10^6^, OPG)	*p* value	
*E. tenella*	NSLC-PLGA	122.923 ± 22.402	0.6446	37.655 ± 7.341	<0.0001	0.889 ± 0.253	<0.0001	86.315
	Blank	117.565 ± 28.329	–	21.599 ± 6.616	–	6.497 ± 1.345	–	–
*E. necatrix*	NSLC-PLGA	119.374 ± 26.856	0.8387	39.327 ± 7.097	<0.0001	0.882 ± 0.111	<0.0001	88.183
	Blank	122.075 ± 31.445	–	22.545 ± 3.937	–	7.466 ± 1.457	–	–
*E. maxima*	NSLC-PLGA	124.445 ± 17.063	0.3584	39.882 ± 7.006	0.0012	1.990 ± 0.657	<0.0001	74.650
	Blank	117.112 ± 17.726	–	26.566 ± 8.501	–	7.851 ± 0.842	–	–
*E. acervulina*	NSLC-PLGA	120.356 ± 13.725	0.6377	38.436 ± 8.171	0.0015	1.782 ± 0.592	<0.0001	79.078
	Blank	123.103 ± 11.849	–	25.938 ± 6.671	–	8.518 ± 2.173	–	–

Ten animals in each group were weighed at 14 (before the first immunization), 28 (before challenge), and 35 (seven days after challenge) days of age, and animals in each group were challenged with *E. tenella* at 28 days of age and euthanized seven days later. Intestinal contents were collected to evaluate the cross-protection of NSLC-PLGA nanospheres against four *Eimeria* spp. Each sample was assessed once, and values were estimated by the independent *t* test. Values are shown as the mean ± SD (*n* = 10). *p* values were obtained by comparison with the blank group.

## Discussion

With the regulations against the cage system in Europe [41], the number of laying chickens kept in floor pens is increasing. In addition, cage-free systems are also popular in the USA. Laying hens housed in floor pens are more frequently affected by avian coccidiosis. However, it is known that *Eimeria* spp. could lead to huge financial losses in broiler chickens when compared with the layer strains, and such perception has caused most studies related to avian coccidiosis to focus on broiler chickens. Thus, an efficient vaccine against coccidiosis in laying chickens has become increasingly urgent. In addition, the application of inbred chicken strains could offer opportunities to eliminate host variation in vaccination and *Eimeria* spp. challenge, thereby minimizing the numbers of animals used in evaluating vaccination outcomes and thus simplifying experimental design [42]. However, it has been shown that some inbred chicken strains are more susceptible to *Eimeria* spp. when compared to other strains [43], and the immunoprotection and consequences after *Eimeria* spp. challenge are likely to be different from the hybrid commercial strains. Thus, a commercial hybrid strain (Hy-Line variety W-36) was selected in the present study.

The secondary structure of multiepitope antigens plays an essential role in determining their ultimate structures and functions. Combined with experimental data and mathematical probability, the GOR method was utilized to assess the secondary structure of NSLC protein. The secondary structure is rich in alpha-helices, which can preserve protein structures and mediate interactions with antibodies [44]. Additionally, the alpha-helix plays an important role in resisting conformational variations [45]. The biological function of the protein is determined by the spatial structure [46]; thus, it is important to understand the structures of NSLC proteins and the connections between the structure and the function. The current research employed the I-TASSER server to construct the predicted 3D structure of the NSLC protein. According to the prediction of secondary structure and the 3D structure, multiple random coil structures were detected in the NSLC protein, and the random coil is important in the flexible nature of the protein [47], showing great antigenic potential. We also confirmed that the NSLC protein did not have any transmembrane domains, indicating that this protein might be recognized by antigen-presenting cells (APCs) to initiate strong immunity. Based on the results of the Antigenic online tool and Western blot analysis, the satisfactory antigenicity of the NSLC protein was demonstrated. Furthermore, the allergenicity evaluation demonstrated that the NSLC protein is a nonallergen. On the basis of these results, we used this protein to estimate its immunogenicity and protective efficacy against avian coccidiosis.

Advances in nanotechnology offer the possibility of delivering vaccines. Currently, various techniques have been constructed to synthesize nanospheres [14, 48]. The physical characteristics of nanospheres can be induced by different methods [38, 48], and in current research, a modified double emulsion solvent evaporation technique and ionic gelation technique were developed for PLGA and chitosan nanosphere synthesis. The synthesized NSLC-PLGA and NSLC-CS nanospheres were round in shape, and the surface was rough due to many convex structures. According to previous research, nanospheres with a diameter of 100 nm are easier for HeLa cells to absorb, and the absorbance is 2.5 times higher than that of nanospheres with a diameter of 1000 nm [49]. In the present research, the mean diameters of NSLC-PLGA and NSLC-CS nanospheres were 77.79 ± 7.86 nm and 100.66 ± 15.49 nm, respectively, leading to better absorption in cells. Moreover, NSLC protein exhibited good characteristics in the formulation of NSLC-PLGA and NSLC-CS nanospheres, with EE values of 77.17% and 75.11%, respectively. Based on a similar strategy, PLGA nanospheres whose EE reached 82.40 ± 0.06% were formulated by Huang et al. [14], while the EE of chitosan nanospheres reached 71% in a previous report [50]. Such differences may be associated with different procedures or antigens, and further research should elucidate the effect of antigens on nanosphere synthesis and optimize the procedures.

The critical role of antibodies in the immune system against *Eimeria* spp. has long been demonstrated. As evaluated by a previous study, antigen-specific antibodies can inhibit *Eimeria* spp. directly and prevent attachment to host cells [51]. Similar to IgG in mammals, IgY is mainly secreted by lower vertebrates, such as birds, amphibians, and reptiles. In the present study, increased production of IgY was observed in the sera of all experimental animals. Our data supported the idea that NSLC protein as well as vaccines could generate an immune response, indicating that NSLC protein and vaccines possessed satisfactory immunogenicity. Similar to our results, immunity could be induced by the recombinant pET-32a-EmROM3 protein in chickens [52]. Furthermore, with the increased titres of IgY in immunized animals, our results also suggested that a booster immunity could be elicited by NSLC protein and vaccines.

Cytokines have been shown to be critical in the immunity against *Eimeria* spp. infection [53, 54]. As a marker of Th1 immunity, the increased secretion of Th1 cytokines, such as IFN-γ, plays a key role in resisting *Eimeria* spp. [55]. In this study, increased secretion of IFN-γ was evaluated in all vaccinated chickens after boost immunization, emphasizing that Th1-related immunity was induced. After Th1 cytokines, Th2 cytokines also play an essential role in resisting *Eimeria* spp. [56]. As a typical Th2 cytokine, IL-4 is responsible for regulating humoral immunity [57]. According to previous studies, IL-4 can effectively defend against extracellular pathogens [58], and the phagocytic enhancement of macrophages induced by IL-4 has been shown when exposed to intracellular parasites [59]. The finding in the present study of the significantly promoted IL-4 production implied that IL-4 plays an important role against avian coccidiosis, which coincides with previous reports [60]. By inhibiting the inflammatory response, cytokines secreted by regulatory T cells (Tregs), such as TGF-β and IL-10, can alleviate intestinal injury [61]. Proinflammatory cytokines and cytokines produced by Tregs play an important role in determining the type of immune response during coccidiosis [62], and the increased TGF-β is beneficial for the recovery of intestinal injury after infection with *Eimeria* spp. [53]. In this study, statistically significant stimulated TGF-β in the sera of experimental animals was detected, implying a benefit in anti-*Eimeria* infections.

Three IL-17 s (IL-17A, Il-17D, and IL-17F) that are secreted by Th17 cells and involved in anti-*Eimeria* infections have been identified in birds [63]. As assessed by quantitative PCR, IL-17A mRNA levels are generally promoted in intestinal intraepithelial lymphocytes (IELs) after a primary infection with *E. acervulina* or *E. maxima* [64]. The maximum expression of IL-17A was 2020 on day five after *E. acervulina* infection and 1650 on day four after *E. maxima* infection. All these results suggest that the expression of IL-17A is associated with *Eimeria* spp. as well as the postinfection time. In the present study, increased IL-17 was observed in partially immunized animals at 14 and 21 days of age, exhibiting a crucial role in immunity against *E. tenella*. Similarly, recombinant *E. tenella* microneme protein 2 (EtMIC2) could induce the expression of IL-17A in chickens, and covaccination of EtMIC2 and *E. tenella* heat shock protein 70 (EtHSP70) could further increase the IL-17A levels compared with single antigen vaccination [65].

Cellular immunity confers a dominant role against *Eimeria* infections [54], and both CD4^+^ and CD8^+^ T lymphocytes are involved. In resisting *Eimeria* spp., CD8^+^ T lymphocytes act as effector cells and can secrete cytokines, while CD4^+^ T lymphocytes are mainly responsible for generating T helper cytokines [56]. Furthermore, CD4^+^ T lymphocytes play a critical role in memory CD8^+^ T lymphocyte formulations after vaccination [66]. The bursa and spleen are immune organs against coccidiosis. Thus, splenic lymphocytes were isolated to analyse the proportions of CD4^+^ and CD8^+^ T lymphocytes after vaccination. In the present study, the proportions of CD4^+^ and CD8^+^ T lymphocytes were obviously promoted in all immunized animals, except for the animals immunized with NSLC protein. These findings made it clear that the NSLC protein entrapped in adjuvants or nanospheres was critical in the formulations of CD4^+^ and CD8^+^ T lymphocytes, which could mediate cellular and humoral immunity against *Eimeria* infection.

Coimmunization with antigens and adjuvants can strengthen induced immunity [67]. When isolated from the root of a ginseng plant, ginsenosides are able to enhance both cellular and humoral immunity against various infections [67], and a previous study proved their enhanced efficiency with recombinant *E. tenella* profilin antigens [68]. A similar result was also reported by Huang et al. in chickens immunized with PLGA nanospheres encapsulated with recombinant *E. tenella* TA4 antigens [14]. Many referenced studies are independent, and the immunoprotection of different adjuvants has not yet been compared. To investigate the optimum adjuvant, NSLC proteins were entrapped in five adjuvants and two nanospheres. In comparison with the neutralized animals, animals from all immunized groups presented significant inhibition of oocyst burden and promotion of the growth coefficient. Among them, NSLC-PLGA nanospheres were effective in oocyst burden inhibition and highlighted their great potential in promoting growth. These results indicated that good immune protection against *E. tenella* could be induced by NSLC-PLGA nanospheres. For the quantification of endoparasite burdens, the most employed methods are to investigate the parasite burden by the analysis of faecal samples and to determine parasite propagules [69]. In *Eimeria* spp., both the excreted faeces [70, 71] and the intestinal contents [13, 72, 73] could be used for quantification of the oocyst burden, and a published paper suggested that faeces originating from the intestine were superior for oocyst quantification [69]. Thus, the intestinal contents were selected for oocyst burdens. For the challenge doses, a wide numerical range should be examined to estimate the vaccine efficacy, with low doses (approximately 100–250 oocysts) to investigate parasite replication and high doses (approximately 5000–50 000 oocysts) to evaluate the immunoprotection [42]. In the current study, only a high-dose challenge was conducted to evaluate the vaccine efficacy, and future studies should reveal the protective immunity of NSLC-PLGA nanospheres in response to a low dose of *Eimeria* spp. challenge.

Attempts should be made to improve the immunity induced by vaccines [73, 74]. Generally, vaccines are administered by the intramuscular, intraoral, and intramucosal routes, but the mechanisms of these routes are still unclear. Even so, attempts to obtain the optimum immune route never stop. For the DNA vaccine against *Eimeria* spp., the intramuscular route is the optimal route among the subcutaneous, intraoral, intravenous, intramuscular, and intranasal routes [74]. Similar results were also obtained for a DNA vaccine expressing *E. tenella* pEtK2 plus chicken IL-2 antigens [73]. However, the intranasal route was shown to be effective for ISCOM immunization in resisting *E. tenella* [75]. Furthermore, compared with the subcutaneous route, the recombinant Bacille Calmette-Guerin vaccines pMV361-rho and pMV361-rho-IL2 could generate robust immunoprotection against *E. tenella* through the intranasal route [76]. In the present study, the intramucosal route was achieved through the nose and eye drops. With a significantly decreased oocyst burden and growth promotion, strong immune protection against *E. tenella* was observed through the intramucosal route. To some extent, our results were consistent with previous studies that proved the intranasal route to be the optimum immune route.

Numerous vaccines have been reported to resist coccidiosis, but no study has explained why a certain immune dosage was used. Understanding the optimum immune dosage is important for making advisable decisions on the recommended immunization strategy. According to the oocyst burdens and coefficients of growth illustrated in the current study, a linear relationship did not exist between immunoprotection and immune dosage. Based on previous studies, the best immune dosage for DNA vaccines against *Eimeria* spp. ranged from 25 to 200 μg [74, 77, 78]. In addition, the optimum immune dosage for the DNA vaccine expressing pEtK2-IL-2 was 80 μg, and it did not show a linear dependence [73]. Due to the different types of vaccines, the obtained immunoprotection varied from one study to another. Furthermore, the effects of immune dosage on multiepitope vaccines have not been described. Thus, a systematic study should be carried out in the future to shed light on subsequent research.

Due to its high pathogenicity and wide prevalence, *E. tenella* is considered the most harmful species among other *Eimeria* spp. [79]. Thus, the animals in previous trials were challenged with *E. tenella* to investigate the optimum adjuvant, immune route, and immune dosage. However, avian coccidiosis is generally caused by several *Eimeria* spp. under natural conditions [1]; thus, an ideal vaccine should provide effective protection against *Eimeria* spp. coinfections. With the oocyst burdens and growth coefficients presented in the present study, a satisfactory protective efficacy could be generated in resisting *E. tenella*, *E. acervulina*, *E. necatrix*, or *E. maxima*, indicating that the NSLC-PLGA nanospheres could be used for preventing the coinfection of avian coccidiosis. Similar results were also confirmed in the combined immunization of four recombinant antigens [15] and recombinant EtMIC2 plus EtHSP70 [65], as well as multiepitope DNA vaccines [80].

In conclusion, our study suggested that the multiepitope vaccine containing B- and T-cell epitopes of *E. necatrix* NA4, *E. tenella* SAG1, *E. acervulina* LDH, and *E. maxima* CDPK had a satisfactory capability of stimulating cellular and humoral immunity against coccidiosis in laying chickens. Among the seven tested adjuvants or nanospheres, PLGA nanospheres loaded with NSLC protein enhanced immune protection and efficacy and were regarded as the optimal vaccine in our studies. In addition, chickens vaccinated with NSLC-PLGA nanospheres containing 300 μg of NSLC protein through the intramucosal route could obtain optimum immunity. However, only the laying chickens (Hy-Line variety W-36) were tested in the current research, and subsequent studies should determine its immunoprotective effects on other chicken breeds. In addition, only partial protection can be elicited by NSLC-PLGA nanospheres, and further studies on this nanovaccine should evaluate the protective immunity under a low dose of challenge, emphasize its immunization strategy, avoid the mortality of infected animals, and enhance immune protection to minimize economic losses.

## Supplementary Information

**Additional file 1. Analysis results of the amino acid sequences of EnNA4 (A), EtSAG1 (B), EaLDH (C), and EmCDPK (D) using DNAStar Protean software.** The amino acids in the red box were selected to construct the fusion protein.

**Additional file 2. Prediction of NSLC protein by GOR IV.** (A): Secondary structure of NSLC protein. h represents an alpha-helix, e represents an extended strand, and c represents a random coil. (B): The graphical results for the secondary structure of the NSLC protein. Blue, red, and purple represent the distributions of the alpha-helix, extended strand, and random coil, respectively.

**Additional file 3. Transmembrane domain prediction of the NSLC protein.**

**Additional file 4. Primers used for PCR amplification.**

## Data Availability

All data generated or analysed in this research are included in this paper and its additional information files.

## Abbreviations

3D: three-dimensional
ANOVA: one-way analysis of variance
APCs: antigen-presenting cells
CDPKs: calmodulin-domain protein kinases
CDS: conserved domain sequence
CFA: complete Freund’s adjuvant
DCM: dichloromethane
EaLDH: *E. acervulina* lactate dehydrogenase
EE: encapsulation efficiency
ELISA: enzyme-linked immunosorbent assay
EmCDPK: *E. maxima* calmodulin-domain protein kinase
EnNA4: *E. necatrix* NA4 antigen
EtHSP70: *E. tenella* heat shock protein 70
EtSAG1: *E. tenella* surface antigen 1
GOR: Garnier-Osguthorpe-Robson
GRAs: dense granules
IFN-γ: interferon-gamma
IL: interleukin
IPTG: isopropyl-β-d-thiogalactopyranoside
ISCOM: immune-stimulating complex
LDHs: lactate dehydrogenases
MICs: micronemes
OPG: oocysts per gram
PLGA: poly lactic-co-glycolic acid
PVDF: polyvinylidene difluoride
ROPs: rhoptries
RT–PCR: reverse transcription PCR
SAGs: surface antigens
SD: standard deviation
SDS–PAGE: sodium salt polyacrylamide gel electrophoresis
SEM: scanning electron microscope
TGF: transforming growth factor
TMB: 3,3′,5,5′-Tetramethylbenzidine
TPP: tripolyphosphate
Tregs: regulatory T cells
