# Supplementary figures and images for: A multiepitope vaccine encoding four Eimeria epitopes with PLGA nanospheres: a novel vaccine candidate against coccidiosis in laying chickens
Source: Vet Res. 2022 Apr 1;53:27. doi: 10.1186/s13567-022-01045-w (PMC9350682; doi:10.1186/s13567-022-01045-w)

| 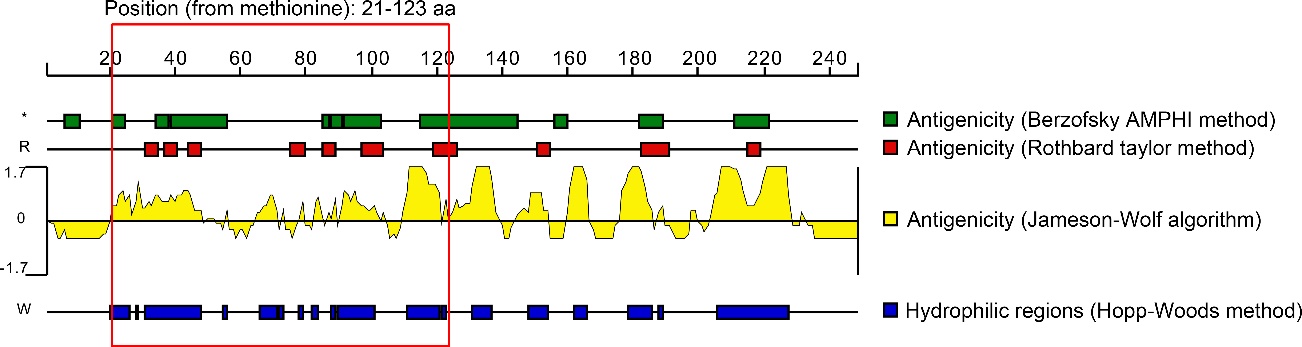 |
| --- |
| (A) |
| 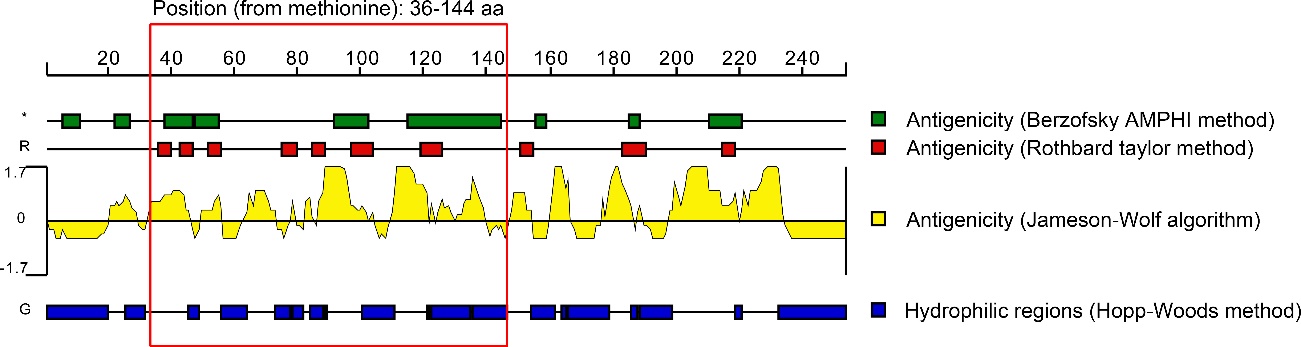 |
| (B) |
| 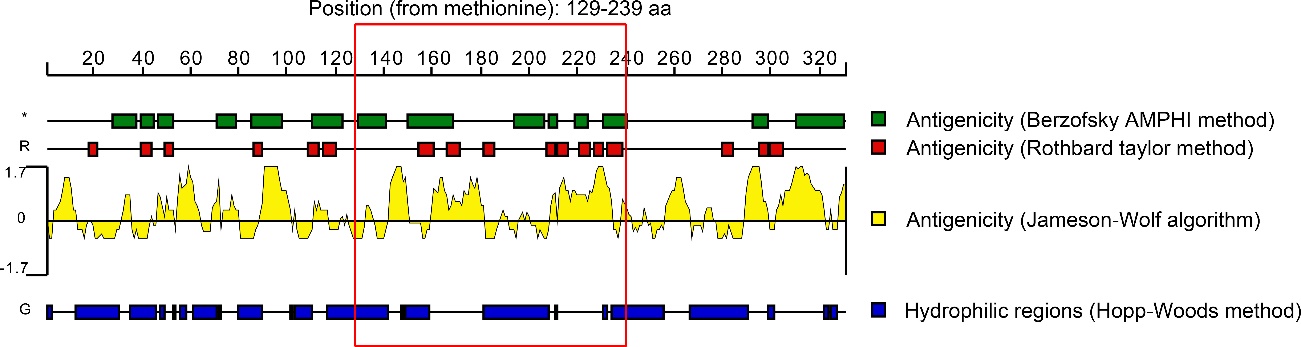 |
| (C) |
| 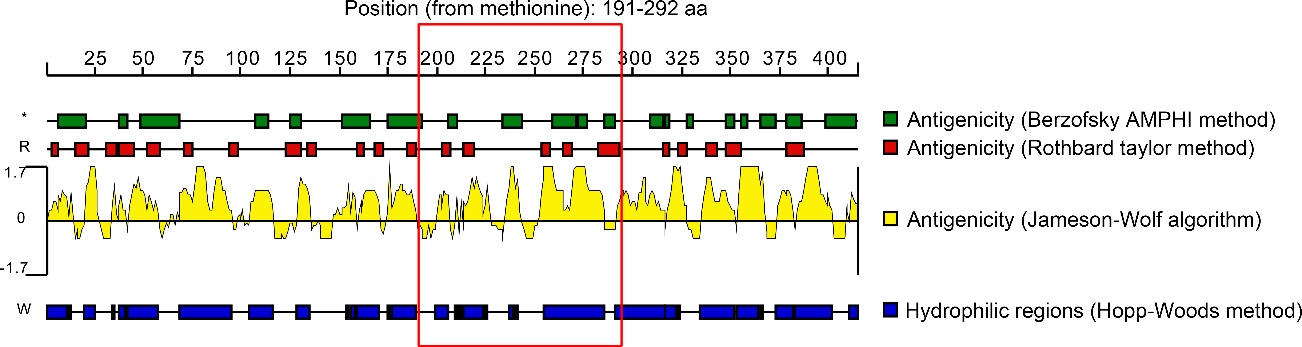 |
| (D) |

Supplement: Supplementary file 1 — Additional file 1. Analysis results of the amino acid sequences of EnNA4 (A), EtSAG1 (B), EaLDH (C), and EmCDPK (D) using DNAStar Protean software. The amino acids in the red box were selected to construct the fusion protein. [file 13567_2022_1045_MOESM1_ESM.docx]

| 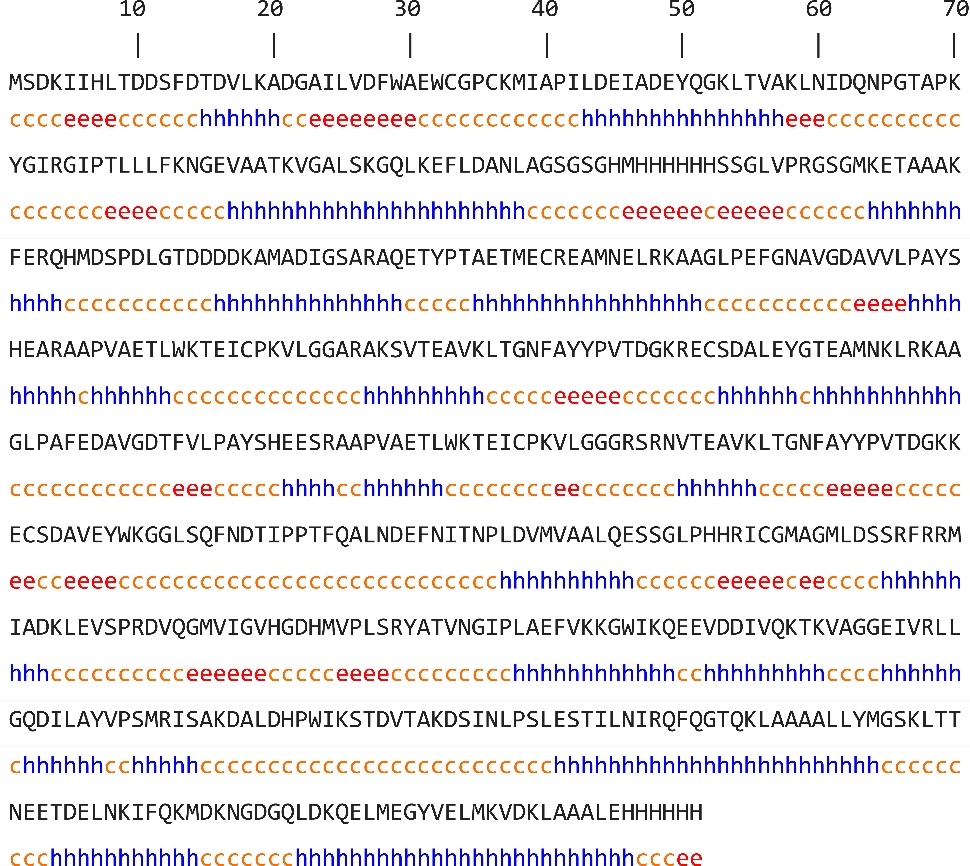 |
| --- |
| (A) |
| 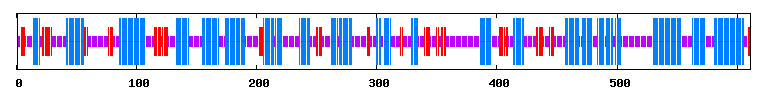  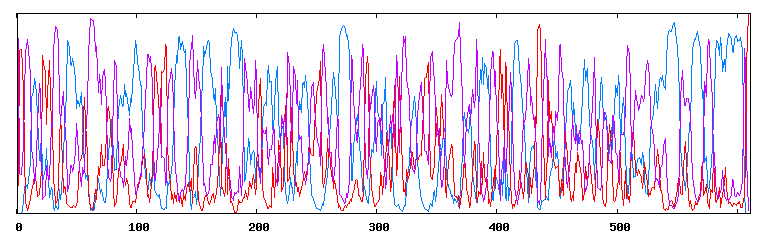 |
| (B) |

Supplement: Supplementary file 2 — Additional file 2. Prediction of NSLC protein by GOR IV. (A): Secondary structure of NSLC protein. h represents an alpha-helix, e represents an extended strand, and c represents a random coil. (B): The graphical results for the secondary structure of the NSLC protein. Blue, red, and purple represent the distributions of the alpha-helix, extended strand, and random coil, respectively. [file 13567_2022_1045_MOESM2_ESM.docx]

| 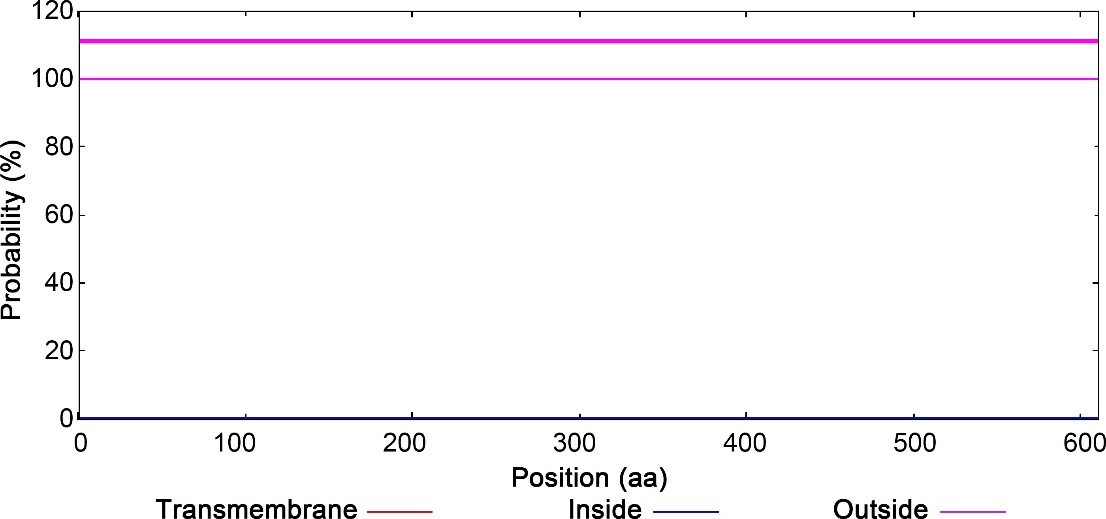 |
| --- |

Supplement: Supplementary file 3 — Additional file 3. Transmembrane domain prediction of the NSLC protein. [file 13567_2022_1045_MOESM3_ESM.docx]
