# Supplementary material for: A multiepitope vaccine encoding four Eimeria epitopes with PLGA nanospheres: a novel vaccine candidate against coccidiosis in laying chickens
Source: Vet Res. 2022 Apr 1;53:27. doi: 10.1186/s13567-022-01045-w (PMC9350682; doi:10.1186/s13567-022-01045-w)
Supplement: Supplementary file 4 — Additional file 4. Primers used for PCR amplification. [file 13567_2022_1045_MOESM4_ESM.docx]

| Name | Sequence (5’-3’) | Restriction endonuclease site | Annealing temperature (°C) | Amplified fragment length (bp) |
| --- | --- | --- | --- | --- |
| EnNA4-F | CGC GGATCC GCCAGAGCTCAGGAAAC | *Bam*HI | 48.7 | 309 |
| EnNA4-R | CGG GGTACC GTACTCCAGAGCATCGC | *Kpn*I |  |  |
| EtSAG1-F | CGG GGTACC GAAGCGATGAACAAGCTG | *Kpn*I | 51.9 | 327 |
| EtSAG1-R | CCG GAATTC GTCGTTCAACGCTTGGA | *Eco*RI |  |  |
| EaLDH-F | CCG GAATTC  AATATAACAAATCCTTTAGAT | *Eco*RI | 42.9 | 333 |
| EaLDH-R | CG GATATC  TTGTCCTAATAGGCGT | *Eco*RV |  |  |
| EmCDPK-F | CG GATATC  AATATAACAAATCCTTTAGA | *Eco*RV | 41.1 | 306 |
| EmCDPK-R | GC GTCGAC CTTCATTAATTCAACATAA | *Sal*I |  |  |
